# PIMD: An Integrative Approach for Drug Repositioning Using Multiple Characterization Fusion

**DOI:** 10.1016/j.gpb.2018.10.012

**Published:** 2020-10-17

**Authors:** Song He, Yuqi Wen, Xiaoxi Yang, Zhen Liu, Xinyu Song, Xin Huang, Xiaochen Bo

**Affiliations:** Department of Biotechnology, Beijing Institute of Radiation Medicine, Beijing 100850, China

**Keywords:** Drug repositioning, Drug similarity network, Multiple characterization fusion, Network pharmacology, Drug discovery

## Abstract

The accumulation of various types of drug informatics data and computational approaches for **drug repositioning** can accelerate pharmaceutical research and development. However, the integration of multi-dimensional drug data for precision repositioning remains a pressing challenge. Here, we propose a systematic framework named PIMD to predict drug therapeutic properties by integrating multi-dimensional data for drug repositioning. In PIMD, drug similarity networks (DSNs) based on chemical, pharmacological, and clinical data are fused into an integrated DSN (iDSN) composed of many clusters. Rather than simple fusion, PIMD offers a systematic way to annotate clusters. Unexpected drugs within clusters and drug pairs with a high iDSN similarity score are therefore identified to predict novel therapeutic uses. PIMD provides new insights into the universality, individuality, and complementarity of different drug properties by evaluating the contribution of each property data. To test the performance of PIMD, we use chemical, pharmacological, and clinical properties to generate an iDSN. Analyses of the contributions of each drug property indicate that this iDSN was driven by all data types and performs better than other DSNs. Within the top 20 recommended drug pairs, 7 drugs have been reported to be repurposed. The source code for PIMD is available at https://github.com/Sepstar/PIMD/.

## Introduction

Despite the ever-increasing funding of pharmaceutical research and development (R&D), the number of new drugs approved has not increased significantly [1]. Traditional *de novo* drug development remains costly, risky, and time-consuming [2], [3]. Drug repositioning, wherein an existing drug receives a new application, provides a new opportunity for pharmaceutical R&D [4]. With the accumulation of drug informatics datasets, computational algorithms can be used for systematic identification of potential new indications for on-market drugs, thereby reducing the financial and time investment, as well as the risk, involved in pharmaceutical R&D [5], [6], [7], [8].

Recent studies have shown that computational approaches based on drug similarity have the potential to reveal novel indications for on-market drugs. These approaches have been applied primarily in the following four ways. 1) The first group includes transcriptional response-based approaches. For instance, Iorio et al. [9] used drug-specific response profiles based on the Connectivity Map (CMap) database to find drugs with similar modes of action, whereas Xie et al. [10] used drug perturbation profiles from the Library of Integrated Network-based Cellular Signatures (LINCS) project to predict additional therapeutic properties of drugs. 2) The second group includes chemical structure-based approaches. For example, Keiser et al. [11] predicted potential drug targets based on combined drug–target structure. 3) The third group includes side effect- or other phenotype-based approaches. For example, Campillos et al. [12], [13] constructed a side effect-driven drug similarity network (DSN) based on the assumption that drugs with similar side effects may share targets leading to the identification of novel drug targets. 4) The last group includes target property-based approaches. For example, Yildirim et al. [14] used known drug–target associations to assess ongoing trends and shifts in drug discovery and to quantify interrelationships between drug targets and disease-causing gene products. Although they shared the assumption that similar drugs tend to share therapeutic properties, each of these studies focused on a single drug feature in assessment of drug similarity, raising some doubts about the usefulness of these approaches. For example, Yildirim et al. [14] pointed out that most drugs with the same targets have different chemical structures, and Keiser et al. [15] demonstrated that a small change in drug structure could alter binding affinity dramatically. In addition, the transcriptional response to drug perturbation may differ across cell lines and drug dosages, thus introducing noise into drug repositioning strategies based on transcriptome data. Notwithstanding, our previous studies illustrated positive correlations between repositioning potential based on transcriptome data and that based on side effect profile or structure [10].

With the development of network pharmacology and systems biology, integrating multi-attribute data of drugs seems to be a feasible means of identifying new opportunities for drug repositioning [16]. One of the most common methods to integrate such data is to concatenate several measurements from various properties, such as side effects and chemical fragments, of each drug [17], [18]. However, the already low signal-to-noise ratio in each data type could be diluted by concatenation [19]. To avoid this problem, many researchers have made some preliminary attempts to use DSNs based on different drug properties for data combination. For example, Napolitano et al. [20] constructed three DSNs, based on drug structures, distances between drug targets in protein–protein interaction (PPI) networks, and expression patterns of drug perturbations, separately. They then integrated these attribute datasets by averaging three drug similarity measurements to predict new therapeutic properties of drugs. Meanwhile, Wang et al. [21] proposed a new algorithm, called PreDR, which predicts as yet unidentified drug–disease associations by taking the maximums of three drug similarity matrices derived from chemical structure, target protein sequence, and side effect profile similarities. Zhang et al. [22] proposed the Similarity-based LArge-margin learning of Multiple Sources (SLAMS) algorithm of drug similarity based on multiple sources of drug and disease property data. SLAMS outputs therapeutic scores for each drug–disease pair that correspond to multi-level drug properties and disease properties, and then averages the scores to predict the novel disease applications for drugs. Liu et al. [23] proposed the two-pass random walks with restart on a heterogeneous network (TP-NRWRH) to predict new indications for approved drugs. In the model, DSNs are integrated using the probability disjunction formula. Additionally, there are many articles on the drug–target predictions with data integration based on a linear combination of multiple attributes [24], [25], [26], [27], [28], [29], [30], [31], [32], [33], [34], [35], [36], [37], [38], [39], [40], [41], [42]. Although many studies take into account multiple drug sources, these integration strategies based on simple averaging or maximization are linear and cannot make full use of topology and non-linear information. Furthermore, most of the methods cannot evaluate the contribution and the relative importance of each property data. As for network-based analysis, to our knowledge, there is no integrated method offering a systematic way to annotate and evaluate the drug clusters. In recent years, non-linear multi-dimensional data fusion algorithms and tools have been widely applied in disease subtype identification, such as the Similarity Network Fusion (SNF) and Integrated Clustering of Multidimensional biomedical data (ICM) [19], [43].

In this study, we propose a systematic and extensible paradigm of drug repositioning, namely prediction of drug therapeutic property by integrating multi-dimensional data (PIMD), and report the construction of an integrated DSN (iDSN) based on drug structure, side effect profile, and target protein sequence data. First, we integrated different types of drug information, including side effects, chemical structures, and molecular targets representing clinical, chemical, and pharmacological properties, separately, and constructed a DSN for each of these properties. Second, we used a non-linear fusion algorithm, namely SNF, to combine three DSNs into the iDSN iteratively [19]. Next, we evaluated the contributions of each dimension of data in the iDSN and the correlation among them.

Our study examined the types of data underlying the iDSN and how the iDSN performs relative to single-property networks. We used spectral clustering to divide the iDSN into clusters, and then conducted a systematic and comprehensive drug cluster analysis through five types of statistical analyses, including drug-based enrichment analysis, target-based enrichment analysis, drug property analysis, chemogenomic enrichment analysis (CGEA), and chemical ontology enrichment analysis. We hypothesize that if similar drugs have similar therapeutic properties, then the drugs that appear unexpectedly in a cluster based on their anatomical therapeutic chemical (ATC) label would represent repositioning candidates.

## Method

### Data source

We collected information on 7132 drugs with their corresponding target protein information from the DrugBank database, a bioinformatics resource with detailed drug data and complete drug–target interaction data. We used version 5.0 of the DrugBank database to construct the DSN based on drug targets (DSN-T) [5]. Protein sequence data were extracted from the UniProt database, which provides high-quality, freely accessible protein sequence data [44].

We obtained 139,756 relationships between 1430 drugs and 5868 side effects from the Side Effect Resource (SIDER) database, which contains information about on-market drugs and their recorded adverse drug reactions or side effects, extracted from public documents and package inserts. We used version 4.1 of SIDER to construct the DSN based on drug side effects (DSN-S) [8].

PubChem Compound is a database containing more than 92 million unique structures of compounds. Similarly, We extracted chemical structures of drugs from PubChem Compound to construct the DSN based on drug chemical structure (DSN-C) [7]. We used the PubChem Compound Identifier (CID) as the only identifier of drugs to identify drugs shared across the databases. We used the identified drugs to construct three single DSNs.

### Drug similarity measurements

Drug similarity quantifies the degree of shared features between paired drugs. We restricted similarity scores to be between 0 (lowest) and 1 (highest). We defined the drug similarity measurements of the three properties examined separately.

#### Drug similarity based on drug side effects

Side effects represent the clinical properties of a drug. We obtained side effect data from SIDER. We used drug side effect information with frequency data (as opposed to without) because such information was derived empirically and thus deemed more credible. Given there are risks of bias in observation and statistics, we filtered outsider effect terms if they occurred only once or with a frequency < 0.1%. Finally, we characterized drug side effects according to the 2072-dimensional binary vector E(d), known as side effect profile. Similarity based on side effects between two drugs d and $d^{\text{'}}$ was computed by the Tanimoto coefficient of their side effect profiles:

(1) $$S_{d,d^{\text{'}}}^{\mathrm{sideeffect}}=\frac{E\left(d\right)\times E\left(d^{\text{'}}\right)}{\left|E\left(d\right)\right|+\left|E\left(d^{\text{'}}\right)\right|-E\left(d\right)\times E\left(d^{\text{'}}\right)}$$

where $\left|E\left(d\right)\right|$ and $\left|E\left(d^{\text{'}}\right)\right|$ are the number of side effect terms for drugs d and $d^{\text{'}}$, respectively. $E\left(d\right)\times E\left(d^{\text{'}}\right)$ represents the number of side effects shared by these two drugs.

#### Drug similarity based on drug chemical structure

Drug chemical structure represents the chemical properties of a drug. We obtained chemical structure information from PubChem Compound and computed atom-pair descriptors of drugs using the R package “ChemmineR” [45]. The atom-pair descriptors used to quantify the chemical structure of small molecule compounds encode all atom pairs in a drug. We computed similarity based on the chemical structure between drugs d and $d^{\text{'}}$ as the Tanimoto coefficient of the chemical atom-pair descriptors:

(2) $$S_{d,d^{\text{'}}}^{\mathrm{chem}}=\frac{C\left(d\right)\times C\left(d^{\text{'}}\right)}{\left|C\left(d\right)\right|+\left|C\left(d^{\text{'}}\right)\right|-C\left(d\right)\times C\left(d^{\text{'}}\right)}$$

where $\left|C\left(d\right)\right|$ and $\left|C\left(d^{\text{'}}\right)\right|$ represent the number of atom pairs for drug d and drug $d^{\text{'}}$, respectively. $C\left(d\right)\times C\left(d^{\text{'}}\right)$ represents the number of the atom pairs shared by these two drugs.

#### Drug similarity based on drug targets

Drug targets represent the pharmacological properties of a drug. We obtained drug–target interactions and target protein sequences from the DrugBank and Uniprot databases, respectively. Then, similarity based on drug targets between drugs d and $d^{\text{'}}$ was computed with a normalized Smith–Waterman score as follows:

(3) $$S_{d,d^{\text{'}}}^{\mathrm{target}}=\frac{1}{\left|P\left(d\right)\right|\times \left|P\left(d^{\text{'}}\right)\right|}\sum_{i=1}^{\left|P\left(d\right)\right|}\sum_{j=1}^{\left|P\left(d^{\text{'}}\right)\right|}\frac{\mathrm{SW}(P_{i}\left(d\right),P_{i}\left(d^{\text{'}}\right))}{\sqrt{\mathrm{SW}(P_{i}\left(d\right),P_{i}\left(d\right))}\sqrt{\mathrm{SW}(P_{i}\left(d^{\text{'}}\right),P_{i}\left(d^{\text{'}}\right))}}$$

where $P\left(d\right)$ represents a target protein set of drug d, $P_{i}\left(d\right)$ indicates the ith target of drug d, and $\left|P\left(d\right)\right|$ represents the size of the target protein set of drug d. $\mathrm{SW}(P_{i}\left(d\right),P_{i}\left(d^{\text{'}}\right))$ is the Smith–Waterman sequence alignment score of target proteins of drugs d and $d^{\text{'}}$ [46].

### SNF method

We used the “SNFtool” in R software to achieve SNF, a useful and popular computational method for data integration in the field of disease subtype identification [19]. It can deal with noise in different data types and make full use of common and complementary information across data types by integrating data in a non-linear way. We introduced SNF into multi-dimensional drug informatics data integration in this study for the first time, in the following three steps. 1) DSNs are built for each data type. 2) Multiple DSNs are integrated with SNF, and each of these DSNs is updated iteratively with information from other networks, making them more similar to each other than before. There are three main parameters in SNF: hyperparameter (η), number of neighbors (K), and number of iterations (T). The integration is robust to these parameters as described previously [19]. Here we set η=0.5, K=20, andT=20, as recommended by Wang and colleagues [19]. 3) A final iDSN is obtained from SNF process convergence.

### Cluster validity index

We used two cluster validity indexes to determine the number of clusters.

#### Dunn index

The Dunn index is the ratio of the smallest distance between observations not in the same cluster to the largest intra-cluster distance. The Dunn index has a value between zero and infinity and should be maximized. The Dunn index is calculated as follows:

(4) $$\mathrm{DI}=\min_{C_{k}\in C}\left(\min_{C_{l}\in C}\frac{\mathrm{dist}(C_{k},C_{l})}{\max_{C_{m}\in C}diam\left(C_{m}\right)}\right)$$

where C is the collection of all clusters, $\mathrm{diam}(C_{m})$ is the largest intra-cluster distance in Cluster $C_{m}$, $whereas\;dist(C_{k},C_{l})$ is the distance between the nearest pair of samples in Cluster $C_{k}$ and Cluster $C_{l}$.

#### Silhouette index

The silhouette value is a measure of how well each object lies within its cluster. The silhouette value ranges from −1 to 1, and should be maximized. It is calculated as follows:

(5) $$S\left(i\right)=\frac{b_{i}-a_{i}}{\max\left(b_{i},a_{i}\right)}$$

where $a_{i}$ is the average distance between sample i and all other data points within the same cluster, $b_{i}$ is the lowest average distance of sample i to all points in any other clusters.

### Evaluation measurements

Several evaluation measurements were used in the study, as introduced below.

#### Normalized mutual information

In probability theory and information theory, the mutual information (MI) of two random variables is a measure of their mutual dependence. In this study, X is the ATC label vector of all the 593 drugs in iDSN, and Y is the predicted label vector obtained by clustering these drugs. MI was calculated as follows:

(6) $$\mathrm{MI}\left(X,Y\right)=\sum_{y\in Y}\sum_{x\in X}p\left(x,y\right)\log_{2}\left(\frac{p\left(x,y\right)}{p\left(x\right)p\left(y\right)}\right)$$

where $p\left(x,y\right)$ is the joint probability function of X and Y, whereas $p\left(x\right)$ and $p\left(y\right)$ are the marginal probability distribution functions of X and Y, respectively. Normalized MI (NMI) is calculated as follows:

(7) $$NMI\left(X,Y\right)=\frac{MI\left(X,Y\right)}{\sqrt{H\left(X\right)\times H\left(Y\right)}}$$

where H(X) and H(Y) are the entropy of X and Y, respectively.

(8) $$H\left(X\right)=-\underset{x\in X}{\sum }p\left(x\right)\log_{2}p\left(x\right)$$

where p(x) is the marginal probability distribution function of X.

#### ATC overlap rate

In drug set D, the number of drug pairs is denoted as *Sum*. For each drug pair $\left(d_{x},d_{y}\right)$ in the set, we calculated the intersection of ATC first-level codes of drugs $d_{x}$ and $d_{y}$. If the intersection was not empty, $AI\left(d_{x},d_{y}\right)=1$, otherwise denoted $AI\left(d_{x},d_{y}\right)=0$. The ATC overlap rate (AOR) in drug set D is computed as follows:

(9) $$AOR\left(D\right)=\sum_{\left(d_{x},d_{y}\right)\in D}\frac{AI\left(d_{x},d_{y}\right)}{\mathrm{Sum}}$$

#### Superclass overlap rate

In drug set D, the number of drug pairs is denoted as *Sum*. For each drug pair $\left(d_{x},d_{y}\right)$ in the set, if the superclass label of drug $d_{x}$ is the same as that of drug $d_{y}$, $SI\left(d_{x},d_{y}\right)=1$, otherwise $SI\left(d_{x},d_{y}\right)=0$. The superclass overlap rate (SOR) in drug set D is computed as follows:

(10) $$SOR\left(D\right)=\sum_{\left(d_{x},d_{y}\right)\in D}\frac{SI\left(d_{x},d_{y}\right)}{\mathrm{Sum}}$$

#### Connectivity

The connectivity indicates the degree of connectedness of the clusters. Denote *N* as the number of observations and denote *C* as the collection of all clusters. *L* represents the number of nearest neighbors. Define ${\mathrm{nn}}_{i\left(j\right)}$ as the j^th^ nearest neighbor of observation i. Let $x_{i,{\mathrm{nn}}_{i\left(j\right)}}$ be zero if *i* and j are in the same cluster, and 1/j otherwise. The connectivity is defined as:

(11) $$\mathrm{Conn}\left(C\right)=\sum_{i=1}^{N}\sum_{j=1}^{L}x_{i,{\mathrm{nn}}_{i\left(j\right)}}$$

The connectivity has a value between zero and infinity and should be minimized.

#### Rogers–Tanimoto index

The Rogers–Tanimoto similarity rely on a 2 × 2 contingency table, consisting of the following four cells: $n_{11}$, $n_{10}$, $n_{01}$, and $n_{00}$. $n_{11}$ is the number of observation pairs, where the two observations belong to the same cluster according to both partition $P_{1}$ and $P_{2}$. $n_{10}$ is the number of observation pairs, where the two observations belong to the same cluster according to partition $P_{1}$ but not to $P_{2}$. $n_{01}$ is the number of observation pairs, where the two observations belong to the same cluster according to partition $P_{2}$ but not to $P_{1}$. $n_{00}$ is the number of observation pairs, where the two observations do not belong to the same cluster according to both partition $P_{1}$ and $P_{2}$. The Rogers–Tanimoto similarity is defined as:

(12) $$\mathrm{RT}=\frac{n_{11}+n_{00}}{n_{11}+n_{00}+2\left(n_{10}+n_{01}\right)}$$

### Other integrative methods for comparison

We compared the network fusion performance of PIMD with three previous integrative methods: 1) the maximum method [21], 2) the weighted average method [20], [22], [24], [25], [26], [27], [28], [29], [30], [31], [32], and 3) the probability disjunction [23]. For the maximum method, we took the maximums of multiple drug similarity matrices. For the weighted average method, we averaged multiple drug similarity matrices by traversing weight. The weight of each drug similarity network is from 0 to 1 with step 0.1. For the probability disjunction, the formula is:

(13) $$S_{d,d^{\text{'}}}=1-\left(1-S_{d,d^{\text{'}}}^{\mathrm{sideeffect}}\right)\left(1-S_{d,d^{\text{'}}}^{\mathrm{chem}}\right)\left(1-S_{d,d^{\text{'}}}^{\mathrm{target}}\right)$$

where $S_{d,d^{\text{'}}}$ is the integrative similarity measurement between drug d and $d^{\text{'}}$.

### Data type contribution

For each edge in the iDSN, we used similarity scores from each single network to describe which data type was the primary contributor. First, we ranked three similarity scores of the edge in each single network as $S_{i}\geq S_{j}\geq S_{k}$, where *i, j*, and *k* refer to the three types of data. If $S_{i}$ was ≥ 10% higher than $S_{j}$, the edge was attributed to the i data type. If $S_{i}$ was < 10% higher than $S_{j}$ but $S_{j}$ was ≥ 10% higher than $S_{k}$, the edge was attributed to both the i and j data types. If $S_{i}$ is < 10% higher than $S_{j}$, but $S_{j}$ is < 10% higher than $S_{k}$, the edge was attributed to all three data types.

### Statistical analysis

We performed five types of enrichment analyses to annotate the drug clusters for drug precision repositioning. For drug-based and target-based enrichment analyses, we calculated enrichment score (ES) as follows:

(14) $$ES=\frac{\mathrm{kN}}{\mathrm{nm}}$$

where k is the number of drugs with a particular label (*e.g.*, ATC code) in the cluster of interest, m is the number of drugs with the label in the overall dataset, n is the total number of drugs in the cluster of interest, and N is the total number of drugs in the overall dataset. Then we used the hypergeometric distribution to calculate the *P* value as follows:

(15) PX≥k=∑x=k∞mxN-mn-xNn

#### Drug-based enrichment analysis

For each cluster, we performed drug class and absorption, distribution, metabolism, excretion, and toxicity (ADMET) property enrichment analyses based on the DrugBank database. We computed ESs and *P* values based on drug ATC code, superclass label, and ADMET properties.

#### Target-based enrichment analysis

For target proteins of each cluster, we performed target class, Kyoto Encyclopedia of Genes and Genomes (KEGG) pathway, and Gene Ontology (GO) enrichment analyses based on the International Union of Basic and Clinical Pharmacology/British Pharmacological Society (IUPHAR/BPS), KEGG, and GO databases, respectively [47], [48], [49]. KEGG and GO analyses were conducted with the ClusterProfiler tool in R software [50]. KEGG and GO enrichment results were selected by a *P* value threshold of 0.05.

#### Drug property analysis

Physicochemical features were extracted from a drug property list in RepurposeDB [51]. Chemical descriptors were computed based on information from the Pybel, JOELib2, and Chemminer chemoinformatics libraries [45], [52], [53]. We used *t*-tests to evaluate the significance of deviation of mean values for each cluster from that of all drugs in DrugBank. In each cluster, for property i in property set I, the deviation from the mean value of all drugs in DrugBank is calculated as follows:

(16) $$D\left(i\right)=\frac{C\left(i\right)-A\left(i\right)}{\left|A(i)\right|},i\in I$$

where $A\left(i\right)$ is the mean property value for all drugs in DrugBank, and $C\left(i\right)$ is the mean property value fro drugs in the cluster.

#### Chemogenomic enrichment analysis

We use the CGEA online tool to analyze drugs in each cluster (http://server.dudleylab.org/index). CGEA maps the drug list to various annotation resources, including drug-induced transcriptional modules, enzymes, and fragments. These potentially relevant features identified provide a rich chemogenomic context for drugs of interest.

#### Chemical ontology enrichment analysis

BiNChE is a web tool for chemical enrichment analysis based on the Chemical Entities of Biological Interest (ChEBI) Ontology [54]. We performed enrichment analysis by mapping the drugs in each cluster to both ‘structure’ and ‘role’ subsets of ChEBI Ontology.

## Results

### An overview of PIMD framework

PIMD is a systematic framework that predicts new therapeutic properties of known drugs, calculates the contributions of various types of data, and annotates various aspects of drug grouping, by integrating multiple drug properties. In this study, we hypothesize that if drug *d_A_* and drug *d_B_* are similar with respect to a particular parameter, then the therapeutic property *t* of drug *d_A_* may also be shared by drug *d_B_*. The functions of a drug can be characterized by multiple drug informatics. Therefore, in PIMD, we investigated three representative drug properties: 1) chemical properties, based on chemical structure data derived from the PubChem Compound database [7]; 2) pharmacological properties, based on protein target sequence data derived from the DrugBank and UniProt databases [5], [44]; and 3) clinical and phenotypic properties, based on side effect data derived from the SIDER database [8].

**Figure 1:**
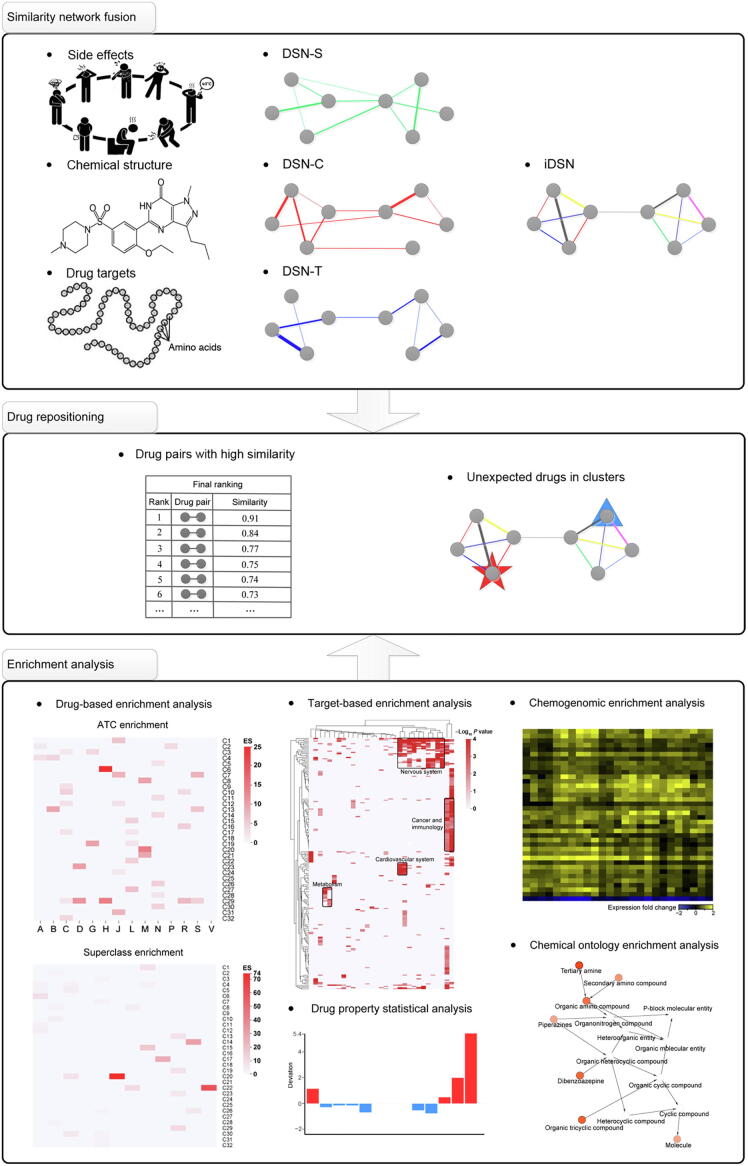
**Construction, drug repositioning, and annotation of iDSN using PIMD** In the first part, we constructed three single-property DSNs. DSNs based on side effects, chemical structure, and drug targets were denoted as DSN-S, DSN-C, and DSN-T, respectively. Then we fused them into the iDSN. In the second part, possible drug repositioning applications were obtained based on high similarity or the occurrence of unexpected drugs within iDSN clusters. Edge color indicates the data type that is the main contributor to the similarity between the drugs. Pentagram and triangle represent unexpected drugs within iDSN clusters. In the third part, 593 drugs were included in the iDSN and grouped into 32 clusters (C1–C32) according to Dunn index and Silhouette values. We then performed a systematic analysis to annotate iDSN clusters from various perspectives to guide drug repositioning. PIMD, prediction of drug therapeutic property by integrating multi-dimensional data; DSN, drug similarity network; iDSN, integrated DSN; ATC, anatomical therapeutic chemical; A, alimentary tract and metabolism; B, blood and blood-forming organs; C, cardiovascular system; D, dermatological; G, genito-urinary system and sex hormones; H, systemic hormonal preparations, excluding sex hormones and insulins; J, anti-infectives for systemic use; L, antineoplastic and immunomodulating agents; M, musculoskeletal system; N, nervous system; P, anti-parasitic products, insecticides, and repellents; R, respiratory system; S, sensory organs; V, various.

The PIMD consists of three parts (Figure 1). In the first part, we constructed three DSNs based on side effects, chemical structure, and drug targets, denoted as DSN-S, DSN-C, and DSN-T, respectively. Then, we applied the SNF method to fuse the DSN-S, DSN-C, and DSN-T into the iDSN in a manner that enables the relative contribution of each data type to be calculated. In the second part, we assessed drug repositioning using two approaches: finding drug pairs with a high similarity score, and identifying unexpected drugs in each cluster. In the iDSN, drug pairs are ranked according to their similarity scores, with highly ranked drug pairs being the most likely to achieve drug repositioning, wherein one drug in a pair may be repurposed for the therapeutic properties of the other drug. We used spectral clustering to distinguish clusters within the iDSN [55], wherein drugs within the same cluster have similar therapeutic properties, as reflected by first-level ATC codes. Within each cluster, drugs with unexpected ATC labels are flagged as having the potential for a repositioning of therapeutic properties. In the third part, aimed at drug precision repositioning, we carried out the following series of enrichment analyses to label and annotate each cluster: 1) drug-based enrichment analysis; 2) target-based enrichment analysis; 3) drug property analysis; 4) CGEA; and 5) chemical ontology enrichment analysis. These analyses can provide guidance to researchers conducting drug repositioning studies from various perspectives. They may also help to elucidate differences in the mode of action of different drug clusters and reveal potential associations between drugs within a cluster.

### Global analysis of iDSN

**Figure 2:**
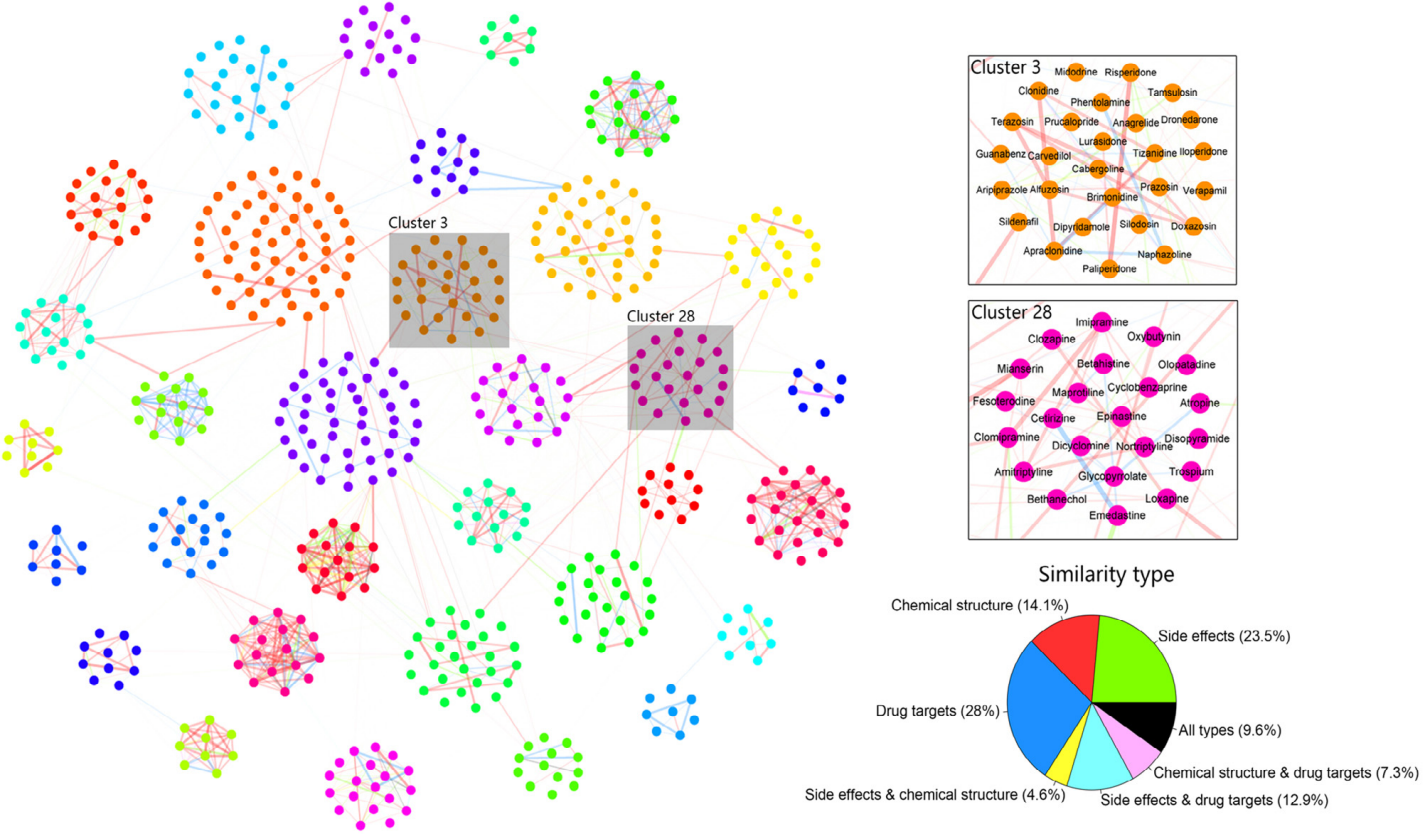
**The iDSN and contributions of various data types** The 593 drugs in the iDSN are grouped into 32 clusters. Each node represents a drug, and node color signifies cluster assignment of the drug. Edges are weighted according to similarity score. Edges are color coded according to the major contributing data types, as defined in the pie chart on the right. Three types of data, including chemical structure, drug targets, and side effects, are used alone or in various combinations to evaluate their respective contributions. Clusters 3 and 28 are zoomed in as examples for better illustration.

We used PubChem CIDs as the sole drug identifiers and extracted chemical structures, target sequences, and side effect sources for 593 drugs from the PubChem Compound, DrugBank, UniProt, and SIDER databases. After constructing the DSN-C, DSN-T, and DSN-S, and combining these three networks into the iDSN with the SNF method, we applied spectral clustering to divide the iDSN into 32 subnetworks (labeled Cluster 1–32) based on two validity indexes (Figure 2, Figure S1) [55], [56].

In the iDSN, each node represents a drug, and the edges connecting the nodes are thickness-weighted according to similarity of the connected drugs. Node color represents the cluster which the drug belongs to. Note that high-similarity connections are found predominantly within clusters. Edge color indicates the data type that is the main contributor to the similarity between the drugs, corresponding to the color scheme in the pie chart. Note that the iDSN model as a whole is supported by all analyzed types of data; in particular, drug pair similarities in the iDSN are supported by two or more types of data, and among the three single drug properties, the highest relative contribution comes from the drug target-based data.

We divided the iDSN edges into two categories: within-cluster edges and between-cluster edges. Data contribution analysis for edges within the cluster (Figure S2) shows that side effect-based data, chemical structure-based data, and drug target-based data account for 11.0%, 22.2%, and 43.9%, respectively. Although the contributions of drug target-based data and side effect-based data are greater than that of chemical structure-based data for all edges in the iDSN (Figure 2), side effect-based data contribute the least to the edges inside the cluster and excessive contribution is from drug target-based data (Figure S2). These results indicate that drug target-based data play a more important role in drug clustering. Data contribution details for each cluster are reported in Table S1. For instance, the contribution of chemical structure-based data in Cluster 6 accounts for 94.4%. We found that Cluster 6 drugs are all peptide drugs, which have chemical structures significantly different from those of other drugs. Cluster 6 drugs also exhibit a significant deviation for physicochemical features (Figure S3A and B). The deviation is consistent with the nature of these peptide drugs.

**Figure 3:**
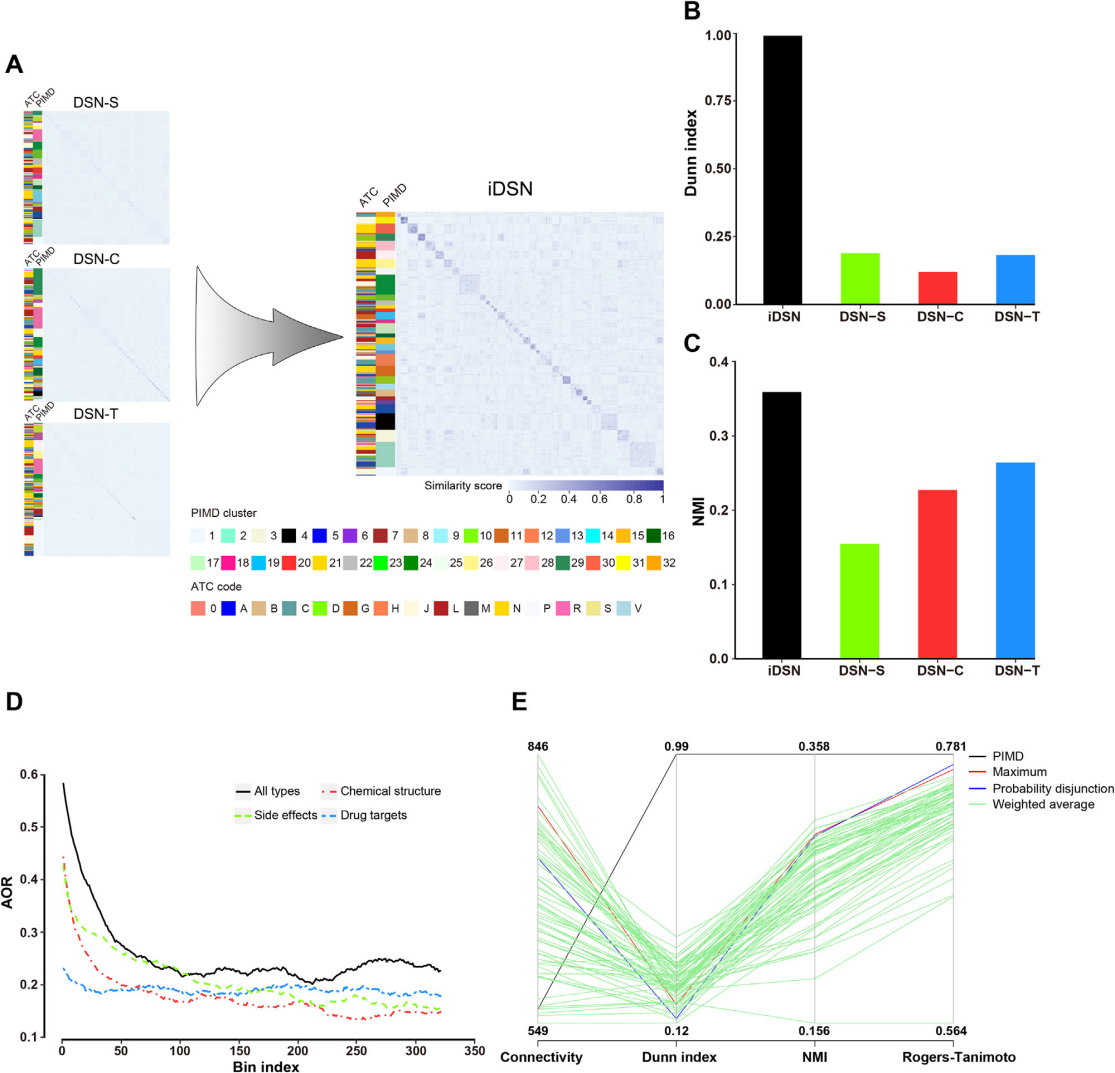
**Performance of the iDSN** **A.** Heatmaps of DSNs. DSN-S, DSN-C, and DSN-T were integrated into the iDSN. The two sidebars to the left of the networks correspond to the original ATC code (left) and PMID cluster label (right) separately. **B.** Dunn index for DSN-S, DSN-C, DSN-T, and iDSN. **C.** NMI for DSN-S, DSN-C, DSN-T, and iDSN. **D.** AOR for DSN-S, DSN-C, DSN-T, and iDSN. A bin composed of 3000 drug pairs was slid from the top to the bottom of the drug pair list with a step size of 100. AOR was calculated for each bin and that for the first 320 bins is plooted here. **E.** PIMD outperforms other integrative methods for DSN clustering. For the weighted average method, multiple drug similarity matrices were averaged by traversing weight. The weight of each DSN ranges from 0 to 1 with the step of 0.1. NMI, normalized mutual information; AOR, ATC overlap rate.

Comparing the performances of the iDSN and each single-property network, we found that the iDSN has a clearer cluster structure than any of the three single-property DSNs (Figure 3A). To verify that, we calculated Dunn index, a metric for evaluating the quality of clustering results. The Dunn index for the iDSN is higher than those for the single-property DSNs (Figure 3B).

Furthermore, compared to the single-property DSNs, the iDSN has a larger overlap between the drug cluster and drug ATC labels. NMI, which reflects consistency across the original ATC and cluster labels, was examined, and a higher NMI for the iDSN was obtained than those for the DSN-C, DSN-T, or DSN-S (Figure 3C). In addition, we compared the AOR of each single-property DSN and the iDSN by first ranking similarity scores of all drug pairs in the iDSN or single-property DSNs and assigning overlapping consecutive drug pair bins with 3000 pairs per bin, such that bin 1 contains the top 3000 most similar drug–drug pairs, bin 2 contains pairs ranked 100th to 3100th in similarity, and so on (first 320 bins are shown in Figure 3D). Subsequent calculation of AORs for each bin shows clearly that the AORs for the iDSN are higher than those of the single-property DSNs (Figure 3D). These results suggest that PIMD makes full use of common and complementary information about drug properties and that the iDSN performs better than any single-property DSNs.

The network fusion of PIMD goes beyond a simple integration representing a maximum or an average of drug similarity measurements. It can capture potential links between drugs. On the one hand, if the similarity score in a single-property DSN is high, but the similarity scores in other single-property DSNs are low, PIMD does not dilute the original information. On the other hand, if the similarity scores for a drug pair in each of the three single-property DSNs are unremarkable, PIMD can still capture potential similarities. For example, the first-level ATC codes for fenoprofen and sulfasalazine are M and A, respectively. Fenoprofen (CID: 000003342) is used for symptomatic relief of rheumatoid arthritis, osteoarthritis, and mild to moderate pain, whereas sulfasalazine (CID: 005359476) is used to treat inflammatory bowel disease. The similarity scores for fenoprofen and sulfasalazine based on the three individual properties are relatively low (rank of 16,951 in DSN-C, 13,466 in DSN-T, and 146,145 in DSN-S), while the iDSN similarity score ranked much higher (rank of 2729) and both drugs were placed in Cluster 8. Although crossover in an application would not have been predicted by any of the single-property DSNs, it has been reported that sulfasalazine can be used to treat rheumatoid arthritis [57], [58].

### PIMD shows better performance than previous integrated methods

To better evaluate the network fusion performance of PIMD, we compared our results with three previous integrative methods: 1) the maximum method [21], 2) the weighted average method [20], [22], [24], [25], [26], [27], [28], [29], [30], [31], [32], and 3) the probability disjunction [23]. Here, we calculated two internal indices (connectivity and Dunn index) and two external comparison indices (NMI and Rogers–Tanimoto index). The internal indices are used to measure the goodness of a clustering structure without external information [59]. The external indices are a measure of agreement between two partitions where the first partition is the *a priori* known clustering structure, and the second partition results from the clustering procedure [60]. The connectivity indicates the degree of connectedness of the clusters. The Rogers–Tanimoto index reflects similarity between the original ATC and cluster labels. Among the four indices, only connectivity should be minimized. We found that PIMD performs the best compared with other methods (Figure 3E). The superior network fusion performance of PIMD results from the application of network-based approach, which is non-linear and utilizes topology information of the network.

### Drug repositioning from two aspects

There are two approaches for drug repositioning: the first is finding drug pairs with a high similarity score; the second is finding unexpected drugs in each cluster. Drug pairs with high similarity in the iDSN could provide us with clues for drug repositioning. The AORs of the top 10 (Table 1) and the top 100 (top 1000 drug pairs are listed in Table S2) drug pairs reached 80% and 94%, respectively. Drug pairs with high similarity scores but different ATC codes may have the potential for repositioning. For example, triptorelin (CID: 025074470) and nafarelin acetate (CID: 025077649) are both gonadotropin-releasing hormone receptor agonists despite having different first-level ATC codes. Interestingly, this drug pair has a high iDSN similarity score and is ranked second in the drug association list. Within the top 20 drug pairs, 7 drugs have been repurposed successfully according to RepurposeDB [51] and Repurposed Drug Database (http://drugrepurposingportal.com/), which record all repurposed drugs thus far. We further checked whether the repositioning in the databases is relevant to our prediction and found that 6 out of these 7 drugs are repositioned for the same purpose as we predicted.

**Table 1:** Top 10 drug pairs in the iDSN

*Note*: Similarity scores between drug pairs among the 593 drugs in the iDSN were calculated using SNF and the 10 drug pairs with highest similarity scores were selected. iDSN, integrated drug similarity network; SNF, Similarity Network Fusion; ATC, anatomical therapeutic chemical; A, alimentary tract and metabolism; C, cardiovascular system; D, dermatological; H, systemic hormonal preparations, excluding sex hormones and insulins; J, anti-infectives for systemic use; L, antineoplastic and immunomodulating agents; M, musculoskeletal system; N, nervous system; R, respiratory system; S, sensory organs; V, various.

Unexpected drugs within an iDSN drug cluster can also signify the potential for repositioning based on distinct therapeutic properties. That is, a drug with an unusual ATC code within a certain cluster may be repositioned for alternative therapeutic properties. The unexpected drugs in each cluster are indicated in Table S3, and some cases are discussed in the section of drug repositioning case using PIMD.

We compared the two drug repositioning approaches together. In the top 100 drug pairs with a high similarity score, there are 6 drug pairs with totally different first-level ATC codes, among which 3 pairs can be discovered using the second approach. In the top 1000 drug pairs, there are 234 drug pairs with totally different first-level ATC codes, among which 75 pairs can be discovered using the second approach. These data indicate a certain overlap between the results predicted using these two approaches. The unexpected drugs in each cluster can achieve drug repositioning between different ATC codes. Compared to the second approach, top-ranking drug pairs with the same ATC codes are more inclined to achieve drug repositioning.

**Figure 4:**
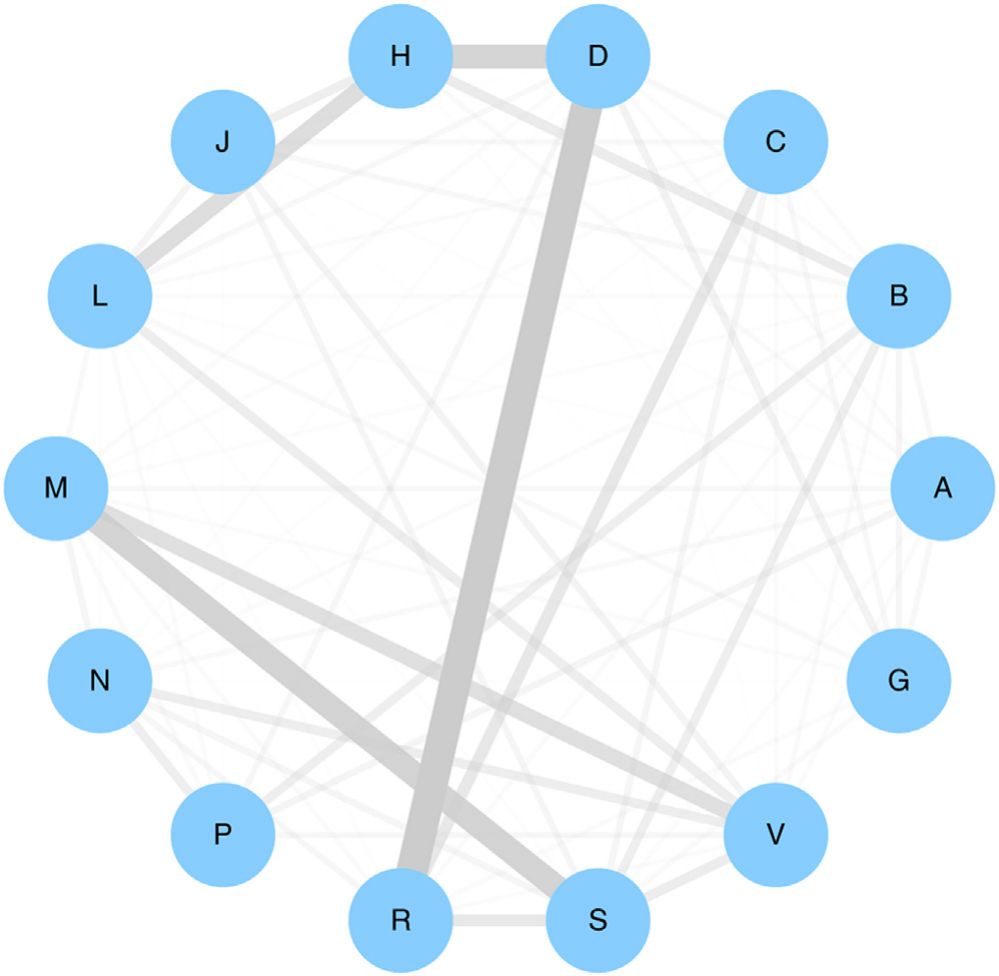
**Global repositioning association among ATC codes** First, we divided drugs found in the top 5% of drug pairs in the iDSN into 14 groups according to their ATC codes. Then we averaged the similarity scores between the different drug groups and used these averages as indices of the repositioning potential within each ATC code. The 14 nodes represent ATC first levels. Edge thickness represents the repositioning potential of drugs between the two ATC codes connected.

To explore the global repositioning association among ATC codes, we analyzed the top 5% of drug pairs in the iDSN. After dividing these drugs into 14 groups according to their ATC codes, we averaged the similarity scores between the different drug groups and used these averages as indices of the repositioning potential within each ATC code. As shown in Figure 4 (where edge thickness represents repositioning potential between two ATCs), there is a particularly high repositioning potential between dermatological drugs and respiratory system drugs. Among 78 dermatological drugs (D) and respiratory system drugs (R), 23 drugs have been repurposed successfully between D and R (Table S4).

### Drug cluster annotation in the iDSN

To label and annotate clusters for drug precision repositioning, we performed iDSN cluster analysis consisting of five statistical analysis methods from drug and target perspectives, thus providing multiple views to verify and select drug clusters for researchers in different fields. These analyses reveal potential links between drugs in the same clusters and differences in the modes of action between drugs in different clusters.

#### ATC, superclass, and ADMET property enrichment analyses of drugs

**Figure 5:**
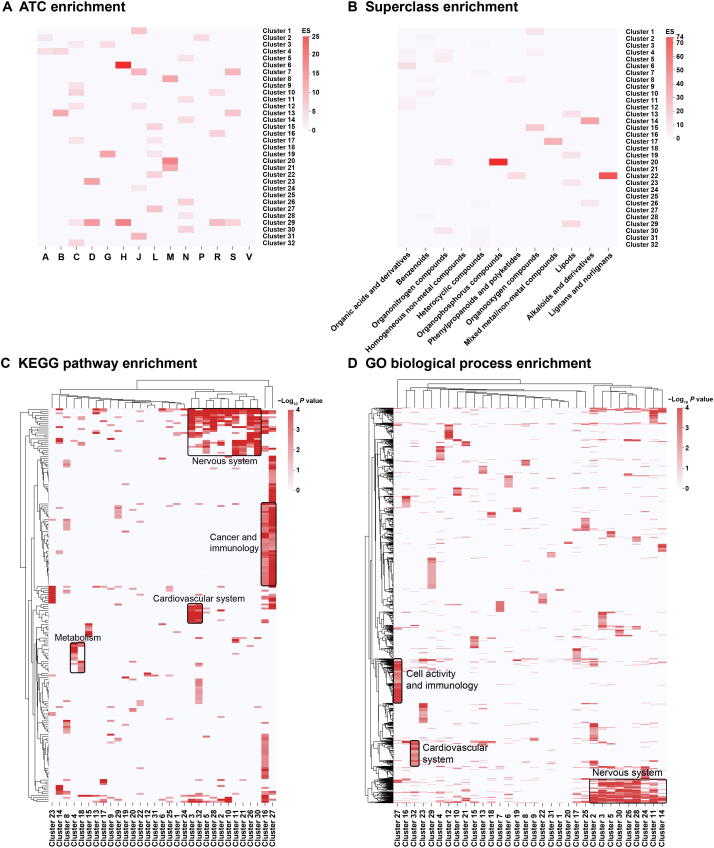
**Drug-based and target-based enrichment analyses** **A.** Enrichment landscape of ATC codes. **B.** Enrichment landscape of superclass codes from the DrugBank database. **C.** Enrichment landscape of KEGG pathways. **D.** Enrichment landscape of GO biological processes. Deeper color signifies greater ES in panels A and B, and signifies greater significance in panels C and D, respectively. Common biological themes shared by multiple clusters are boxed with names provided in the plot. ES, enrichment score; GO, Gene Ontology. Enrichment matrices of ATC codes and superclass codes were obtained using Fisher’s exact test based on hypergeometric distribution. KEGG and GO enrichment results were obtained using the ClusterProfiler tool in R software.

We set to explore to what extent drugs within a cluster share a common ATC code. For each cluster, we computed an ATC code enrichment score and an accompanying *P* value. We found that 30 out of 32 clusters were significantly enriched for at least one ATC code (*P* < 0.05; Figure 5A). Furthermore, 25 out of 32 clusters were significantly enriched for at least one superclass label (*P* < 0.05; Figure 5B). The superclass label of the drug is extracted from the DrugBank database and focuses more on the chemical attributes of drugs. Combining the two enrichment results may lead to new discoveries. For example, Cluster 22 was found to be enriched in the lignan/norlignan superclass as well as in the antineoplastic and immunomodulating agent ATC code. Recent studies have shown that lignans/norlignans play an important role in anti-cancer therapies [61]. In addition, we extracted 18 ADMET property terms from the DrugBank database and examined whether drugs within the same cluster tend to have the same ADMET properties (Figure S3C). For example, 6 clusters are enriched in the nervous system ATC code, while four of them (Cluster 11, Cluster 26, Cluster 28, and Cluster 30) are also enriched in the blood brain barrier (+). This observation is in line with the knowledge that nervous system drugs usually need to penetrate the blood brain barrier.

#### KEGG and GO enrichment analyses of targets

To explore whether drugs in the same cluster tend to target similar proteins and whether particular drug classes are associated with particular target classes, we performed KEGG pathway and GO enrichment analyses for targets of each drug cluster [48], [49], [50]. The most enriched pathways, biological processes, cellular components, and molecular functions (Figure 5C and D, Figure S3D and E; result matrices are listed in Table S5) differ substantially among the different clusters, highlighting differences in mode of action of drugs. Nonetheless, some drug clusters were found to contain common pathways and biological processes. We applied key biological themes for major clusters. For example, Cluster 27, which is enriched for antineoplastic and immunomodulating agents, was most enriched for the KEGG pathways of Rap1 signaling pathway, Ras signaling pathway, and central carbon metabolism in cancer. The most enriched GO biological process terms for Cluster 27 include protein autophosphorylation, positive regulation of MAPK cascade, and phosphatidylinositol-mediated signaling. These pathways and biological processes are indeed closely related to the mechanisms of antineoplastic drugs.

Another example is Cluster 16. Drugs in Cluster 16 are enriched for the ‘respiratory system’ ATC code. Chemical ontology enrichment analysis shows that drugs in this cluster are enriched for methylxanthine. The methylxanthines in Cluster 16, such as caffeine and theophylline, are used in therapy for respiratory diseases. However, the KEGG pathway enrichment analysis shows that drug targets in this cluster are also enriched for some cancer and immunology related pathways. These data suggest the association between methylxanthine and cancer, which is supported by recent studies [62], [63].

Furthermore, we conducted target enrichment analysis for each cluster based on class information. The class information is collected from the IUPHAR/BPS database [47], which indicates the type of drug targets, such as G-protein-coupled receptors (GPCR), catalytic receptor, and enzyme*.* We found that 24 of the 32 iDSN clusters were significantly enriched (*P* < 0.05; Figure S3F). For example, Cluster 32 is enriched in the voltage-gated ion channel (VGIC) target class as well as in the ‘cardiovascular system’ ATC code. This observation is in line with the fact that most of Cluster 32 drugs are calcium channel blockers and used as antihypertensive drugs, whose targets mainly include calcium voltage-gated channel alpha1 (CACNA1) subunits.

#### Physicochemical feature and chemical descriptor analyses of drug properties

Given pharmacological profiling of small molecules may also affect drug repositioning, we analyzed the characteristic physicochemical features and chemical descriptors for each cluster. In total, we extracted 14 physicochemical features from the RepurposeDB drug property list [51] and 62 chemical (*i.e.*, atomic, compositional, and geometric) descriptors. These properties were quantitated using the Pybel, JOELib2, and Chemminer chemoinformatics tools [45], [52], [53]. For each property, we calculated mean values of these drug properties for all drugs in DrugBank and in each cluster. We found that the mean value of drug properties for drugs in each cluster was deviated from that for all drugs in DrugBank (Table S6). The degrees of statistically significant deviation (*P* < 0.05; see Method) are shown in Figure S3A and G. Considering the bias resulting from the incompleteness of drug set, we also compared the mean value of drugs in each cluster with those of all the 593 drugs in the iDSN (Figure S3B and H).

#### Chemogenomic enrichment analysis

CGEA, similar to gene set enrichment analysis, is a method that compares drugs with a range of biological and chemical annotations [64] (http://server.dudleylab.org/index). By identifying chemogenomic characteristics shared by sets of drugs such as enzymes, transporters, and structural fragments, we can obtain abundant chemogenomic context for a biological state of the drug set. We used CGEA to analyze drugs in each cluster for biological and chemical annotations. The results are listed in Table S7. The example of CGEA is described in detail in the section of ‘Case study: drug repositioning using 4 clusters of iDSN’.

#### Enrichment analysis of chemical ontology

Chemical ontology enrichment analysis is based on the ChEBI Ontology, which is a dictionary of chemical compounds with biological roles [65]. Mapping drugs to the ChEBI database can improve our understanding of biochemical nature of drugs. We performed a chemical ontology enrichment analysis of the drugs in each cluster using the BiNChE tool [54]. The resultant ChEBI-based enriched structure terms and role terms for each cluster are provided in Table S8. The example of chemical ontology enrichment analysis is described in detail in the section of ‘Case study: drug repositioning using 4 clusters of iDSN’.

These analyses validate the rationality of the cluster division. Furthermore, by performing the 5 types of analyses described above, we have a comprehensive understanding of various properties of drugs in the cluster to conduct drug repositioning better. For example, Cluster 15 and Cluster 27 are both enriched for antineoplastic and immunomodulating agents, but their enriched KEGG pathways and GO terms (Figure 5C and D, Figure S3D and E) differed from each other. Chemical ontology enrichment analysis shows that Cluster 15 drugs have a role term of antimetabolite, while Cluster 27 drugs have a role term of protein kinase inhibitor. Most of Cluster 15 drugs are purine or pyrimidine analogs, whereas most of Cluster 27 drugs are tyrosine kinase inhibitors. These analyses highlight the differences in mode of action of the drugs. In Cluster 15, ribavirin (ATC code for ‘anti-infectives for systemic use’), which is a guanosine analog used for anti-virus, shows its therapeutic potential for cancers [66], and its mode of action is similar to other Cluster 15 drugs.

Additionally, these analyses provide us a chance to look at the same drug cluster in combination of different perspectives, which may bring us some new discoveries. For example, Cluster 5 drugs are enriched for the ‘nervous system’ ATC code. Chemical ontology enrichment analysis shows that Cluster 5 drugs have a role term of serotonergic drug, a type of nervous system-related drugs. KEGG pathway enrichment and GO enrichment analyses also reveal terms related to the nervous system. However, there are also terms related to cardiovascular system according to cellular components (CC) and molecular function (MF) of GO enrichment analysis results (Figure S3D and E). In ADMET property enrichment analysis, Cluster 5 drugs are enriched in human ether-a-go-go-related gene (hERG) inhibition (predictor I, strong inhibitor) and hERG inhibition (predictor II, inhibitor). hERG inhibition is related to QT prolongation. Moreover, CGEA results indicate that Cluster 5 drugs are enriched for the side effect electrocardiogram QT prolonged. These aforementioned analyses suggest that some serotonergic drugs (especially in Cluster 5) may cause QT prolongation. Indeed, some recent studies revealed the association between serotonergic drugs and QT prolongation [67], [68].

### Case study: drug repositioning using 4 clusters of iDSN

To illustrate the drug repositioning performance of PIMD, we examined some clusters in this section.

**Figure 6:**
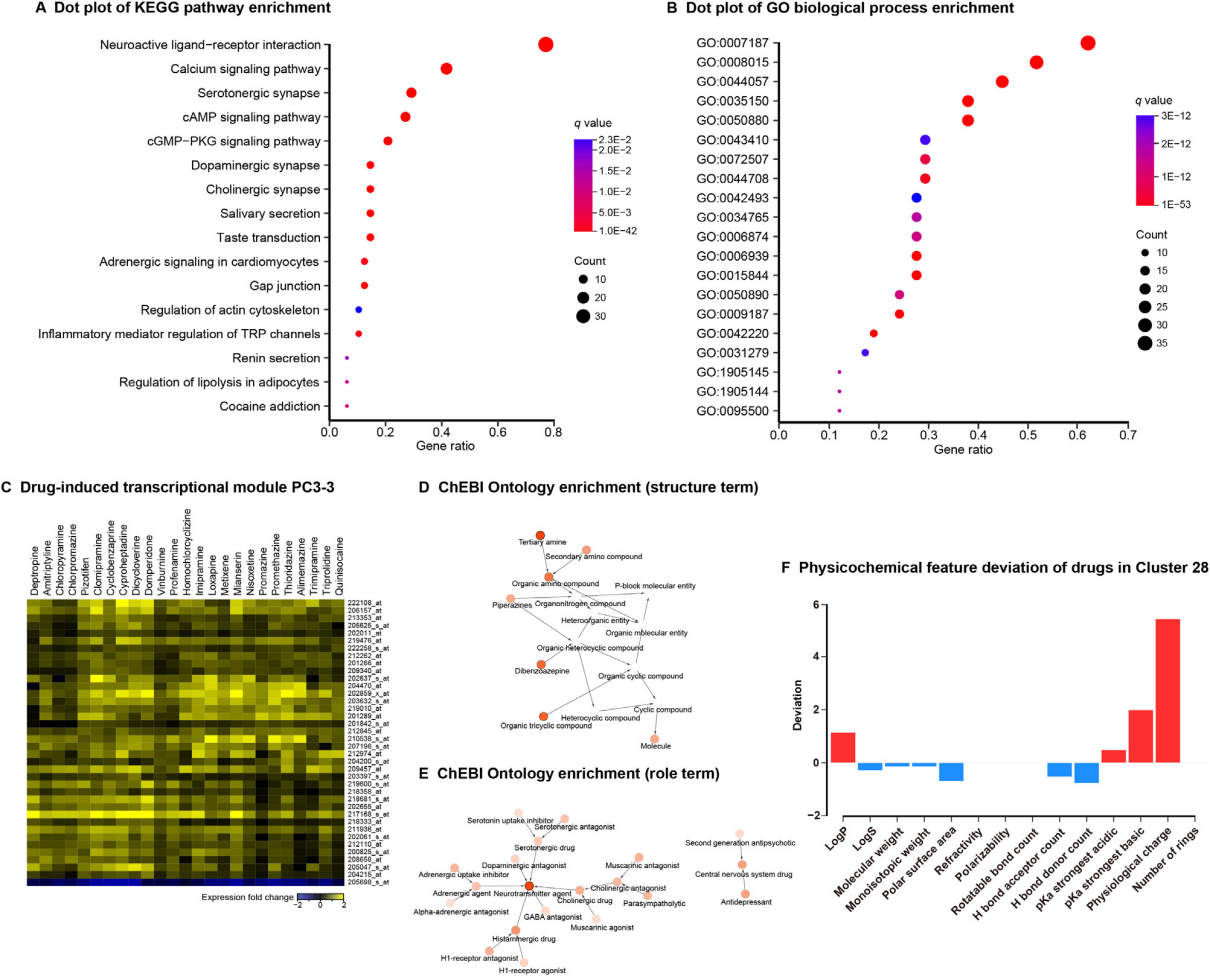
**Community analysis of Cluster 28** **A.** Dot plot of KEGG pathway enrichment results. **B.** Dot plot of GO biological process enrichment results. Dot size and color indicate the count of enriched genes in each of the categories and the corresponding significance of enrichment, respectively. Gene ratio represents the ratio of enriched genes to all genes in each of the categories. GO IDs are presented here for simplicity. The list of corresponding GO terms associated with these IDs can be found in Table S9. **C.** Drug-induced transcriptional module PC3-3. The transcriptional module data were obtained from the study of Iskar and colleagues [69]. Drug enrichment analysis was performed using CGEA. The horizontal axis represents the drug name and the vertical axis represents the gene probe ID, respectively. **D.** ChEBI Ontology structure term enrichment results. **E.** ChEBI Ontology role term enrichment results. Nodes indicate the enriched ChEBI terms. The lower the node transparency is, the more significantly the term is enriched. Arrows proceed from child to parent terms. **F.** Physicochemical feature deviation of drugs in Cluster 28. Red and blue bars represent the indicated drug feature of Cluster 28 that is higher or lower than the average of all drugs in the DrugBank database, respectively. CGEA, chemogenomic enrichment analysis; ChEBI, Chemical Entities of Biological Interest.

The Cluster 28 drugs (Figure 2) are enriched for the ‘nervous system’ ATC code, the ‘benzenoids’ superclass label, and the ‘G-protein coupled receptors’ target class. KEGG pathway enrichment and GO enrichment analysis results also include terms related to the nervous system (Figure 6A and B, Figure S4A and B; Table S9). Our CGEA analysis shows that Cluster 28 drugs are enriched for the molecular fragment CCCN(C)C and drug-induced transcriptional module PC3-3 (Figure 6C). The module contains expression profiles for 38 genes in response to 25 drugs, 6 drugs out of which are included in Cluster 28 [69]. The most prominent drug mode of action among Cluster 28 drugs is ‘Antihistamines for systemic use’, which can affect the central nervous system [70]. Chemical ontology enrichment analysis illustrates that Cluster 28 drugs are enriched with neurotransmitters, neurotransmitter derivatives, and central nervous system drugs (Figure 6D–F; drug property analysis results in Figure 6F). We found that cyclobenzaprine, a drug used to treat skeletal muscle spasms and fibromyalgia, is an unexpected drug in Cluster 28. Cyclobenzaprine, as well as another drug named the anti-depression drug amitriptyline in this cluster, exhibits antagonistic effects on the 5-hydroxytryptamine receptor 2A and possesses a tricyclic structure. Cyclobenzaprine is being studied for the post-traumatic stress disorder treatment according to ClinicalTrials.gov (https://clinicaltrials.gov/), a database of clinical studies conducted around the world. Therefore, the novel therapeutic property for cyclobenzaprine might be N (nervous system). These results indicate that Cluster 28 drugs are related to nervous system and show the good compatibility. Unexpected drugs in Cluster 28 also have the potential to be repositioned to ‘nervous system’ ATC code.

Similarly, pentoxifylline, an unexpected drug in Cluster 16 (drugs in this cluster are enriched for the ‘respiratory system’ ATC code), is an interesting case. Pentoxifylline carries an ATC code for ‘cardiovascular system’ and is used in therapy for intermittent claudication [71]. Notably, it has a high similarity score with theophylline (ATC code for ‘respiratory system’), another drug in Cluster 16 used to treat respiratory diseases such as asthma. They are both members of the xanthine family, so they have a relatively high similarity score based on chemical structure (rank of 2898 in DSN-C). They also have a relatively high similarity score based on drug targets (rank of 7277 in DSN-T) because of the presence of 5 common targets. Pentoxifylline can increase red blood cell deformability and decrease blood viscosity [72]. But its similarity with theophylline has encouraged researchers to explore the potential of pentoxifylline to treat asthma [73].

Clusters simultaneously enriched in two therapeutic properties can also provide a unique perspective for drug repurposing. Cluster 3 drugs (Figure 2) are enriched for both the ‘cardiovascular system’ and ‘genitourinary system and reproductive hormones’ ATC codes (Figure S5A–G; Table S9). In this cluster, iloperidone, a drug used for schizophrenia, may be repositioned for applications related to the ‘cardiovascular system’ and the ‘genitourinary system and reproductive hormones’. Indeed, a previous study showed that repeat administration of iloperidone moderated hypotension [74]. Interestingly, we noted that sildenafil, a drug that was successfully repositioned from ‘cardiovascular system’ treatment to ‘genitourinary system and reproductive hormones’ treatment, is also present in Cluster 3, suggesting that drugs in this cluster may have the potential for both therapeutic properties. Likewise, prazosin and terazosin are an interesting pair. Prazosin (ATC code for ‘cardiovascular system’) is used to treat hypertension and also for urinary hesitancy associated with prostatic hyperplasia [75]. Terazosin (ATC code for ‘genitourinary system and reproductive hormones’) is used to treat enlarged prostate symptoms, which can also moderate hypertension [76], [77]. Hence, the primary indications of these two drugs can be treated as secondary indications of each other. In terms of target proteins, both drugs have antagonistic effects on alpha-1A, -1B, and -1D adrenergic receptors. In terms of chemical structures, their most common substructure is the molecular fragment O=CN1CCNCC1. Their common adverse reactions include dizziness, headache, drowsiness, lack of energy, and weakness.

Another interesting case is cyproterone acetate (ATC code for ‘genito-urinary system and sex hormones’) in Cluster 19. Cyproterone acetate can not only treat androgen-dependent conditions like excessive hair growth and acne but also treat prostate cancer [78]. Therefore, the novel therapeutic property for cyproterone acetate could be L (antineoplastic and immunomodulating agents). More interestingly, cyproterone acetate was originally developed as a progestin [79], but it was first marketed as an antiandrogen [80]. Cluster 19 drugs are enriched for both the ‘genito-urinary system and sex hormones’ and ‘antineoplastic and immunomodulating agents’ ATC codes. In this cluster, cyproterone acetate has high similarity scores with bicalutamide (ATC code for ‘antineoplastic and immunomodulating agents’) and medroxyprogesterone acetate (ATC code for ‘antineoplastic and immunomodulating agents’ and ‘genito-urinary system and sex hormones’). For the drug pair of cyproterone acetate and medroxyprogesterone acetate, their similarity score based on the chemical structure is relatively high (rank of 101 in DSN-C), but the similarity score based on drug targets is relatively low (rank of 35,974 in DSN-T). On the contrary, cyproterone acetate and bicalutamide have relatively high similarity score based on drug targets (rank of 178 in DSN-T) but relatively low similarity score based on chemical structures (rank of 81,257 in DSN-C). This is because cyproterone acetate is more structurally similar to medroxyprogesterone acetate (a type of progestin) but has antiandrogenic effects on androgen receptor like bicalutamide (a kind of antiandrogen). This explains the uniqueness of cyproterone acetate. These results also show that PIMD takes advantage of the universality, individuality, and complementarity of different drug properties.

## Discussion

Here, we proposed PIMD, an integrative, systematic, and extensible framework for discovering novel therapeutic properties of drugs from heterogeneous data sources. PIMD characterizes the iDSN by integrating chemical structure data, target protein sequence data, and side effect data; and performing spectral clustering of the iDSN identified 32 drug clusters. Additionally, PIMD facilitates drug repositioning from two aspects: drug pairs with high iDSN similarity score and unexpected drugs in each cluster. Finally, via a series of enrichment analyses, PIMD annotates and evaluates all clusters from chemical, pharmacological, and genomic views. Thus, PIMD screens suitable clusters for drug precision repositioning.

By integrating multi-dimensional drug informatics data, PIMD can capture potential similarities between drugs with sensitivity. The iDSN is superior to single-property DSNs in multiple evaluation measurements, including AOR, NMI, and cluster compactness. In this study, we primarily used ATC label as a golden standard to annotate drugs and evaluate the performance of PIMD and other compared methods. Interestingly, if the superclass label is used to calculate the evaluation measurements instead of ATC code, the SOR and NMI values of chemical structure-based DSN are higher than those of other DSNs, even the iDSN (Figure S6A and B). This suggests that chemical property has a large positive effect on superclass, whereas clinical and pharmacological properties could affect drug superclass label negatively. Indeed, ATC label and superclass label are two different drug catalogs. ATC label can comprehensively reflect therapeutic properties of drugs, thus commonly used to classify and label drugs, whereas superclass label primarily represents the chemical properties of the drugs. This result can improve our understanding of the difference between ATC and superclass labels of the drugs.

PIMD offers new insights into the universality, individuality, and complementarity of three drug properties, including chemical, pharmacological, and clinical properties. Our calculations of the relative contribution of each data type indicate that the iDSN is driven by all data types. PIMD makes full use of the information on each property. Examination of NMI among single-property, dual-property, and three-integrated-property DSNs (Figure S7A–D) shows that networks based on single property alone overlap marginally but are complementary to each other instead. Furthermore, PIMD provides the drug property contribution to each cluster, improving our understanding of the cluster characteristics (Table S1).

Rather than simple fusion, PIMD provides a systematic way to evaluate the drug cluster and drug repositioning. Pharmacochemistry and pharmacogenomics researchers can use PIMD to screen drug clusters based on their own requirements (Figure 5A–D, Figure S3A–F; Tables S7 and S8).

As a highly extensible framework, PIMD can fuse various properties beyond the three properties examined here. For example, combining drug expression profiles would allow us to elucidate similarities between drugs at the transcriptional level. Drugs have multi-dimensional properties, and drug effects on disease processes are an interdisciplinary issue. The complementarity among multiple properties allows us to assess drug similarities more accurately and thus to provide a more comprehensive and clearer direction for drug repositioning.

There are two reasons why we chose these three data sources as an example. Firstly, these three data sources are representative of a variety of drug informatics data. Side effect, chemical structure, and molecular target data represent the clinical, chemical, and pharmacological properties of drugs, respectively. The three properties comprehensively summarize the drug characteristics. Secondly, these three data sources have sufficient data to extract. We can collect plenty of these data from the public databases. Moreover, despite insufficient data available, we also compared iDSN with DSNs based on three other data types: 1) drug 3D structure, 2) drug expression profiles, and 3) PPI network (similarity measurements can be found in File S1). The results show that iDSN performs best compared with other DSNs (Figure S8).

The current study is limited by the set of drugs available when using the intersection of drug sets with multi-dimensional properties. If a certain property of a drug is not available, it would be excluded from the construction of iDSN. Therefore, with the accumulation of drug informatics data in the future, we expect that the scale of iDSN would be expanded, and the performance of PIMD would be further improved accordingly.

In summary, PIMD provides a new perspective for drug repositioning through multi-property fusion and an analysis package. It facilitates to understand the integration of drug properties at a deeper level, and its high expansibility and modularity would allow users to explore drugs from a wider range of fields.

## Code availability

Source code of PIMD is available at https://github.com/Sepstar/PIMD/.

## CRediT author statement

**Song He:** Conceptualization, Methodology, Investigation, Software, Visualization, Writing - original draft, Writing - review & editing. **Yuqi Wen:** Conceptualization, Methodology, Investigation, Software, Visualization, Writing - original draft, Writing - review & editing. **Xiaoxi Yang:** Investigation, Visualization. **Zhen Liu:** Investigation, Visualization. **Xinyu Song:** Investigation, Visualization. **Xin Huang:** Investigation, Visualization. **Xiaochen Bo:** Conceptualization, Methodology, Supervision, Software, Writing - review & editing. All authors read and approved the final manuscript.

## Competing interests

The authors have declared no competing interests.

## Acknowledgments

This work was supported by the 10.13039/501100001809National Natural Science Foundation of China (Grant No. U1435222) and the Program of International Sci-Tech Cooperation, China (Grant No. 2014DFB30020).

## Supplementary material

Supplementary File S1 Drug similarity measurements.

Supplementary Figure S1 Cluster validity index. The Dunn index is the ratio of the smallest distance between drugs not in the same cluster to the largest intra-cluster distance, ranging from 0 to 1. The Silhouette index is a measurement of how similar a drug is to its own cluster compared to other clusters, ranging from −1 to 1. Both indices should be maximized. Numbers on X-axis are cluster number ranging from 3 to 43, which are set to find the optimal number for clustering.

Supplementary Figure S2 Data type contribution analysis for the edges inside the cluster. In the iDSN, within-cluster edges are supported by all types of data analyzed. Among the three single drug properties, the relative contribution of drug target-based data is the greatest.

Supplementary Figure S3 Series of enrichment analyses. For each property (14 physicochemical features),we determined the deviation of its mean value in each cluster from that for all drugs in DrugBank (**A**) or the mean value for all the 593 drugs in iDSN (**B**). Red and blue blocks represent features in the cluster that are higher and lower than the average of all drugs in DrugBank or in iDSN, respectively. **C**. ADMET enrichment analysis. A total of 18 ADMET property terms were extracted from DrugBank. Each property contains two condition terms. We examined whether drugs in the same cluster tend to have the same ADMET property. The higher the ES is, the deeper the color is. **D**. Enrichment landscape of “target GO cellular component. The deeper the color is, the more significant the enrichment is. Common cellular component themes shared by multiple clusters are boxed with names provided in the plots. **E**. Enrichment landscape of target GO molecular function. **F**. Enrichment landscape of target classes. The higher the ES is, the deeper the color is. Target class: LGIC, ligand-gated ion channels; GPCR, G protein-coupled receptors; VGIC, voltage-gated ion channels; NHR, nuclear hormone receptor; Other IC, other ion channels. For each property (62 chemical descriptors), we determined the deviation of its mean value for drugs in each cluster from the mean value for all drugs in DrugBank (**G**) and the mean value for all the 593 drugs in iDSN (H). Red and blue blocks represent features in the cluster that are higher and lower than average of all drugs in DrugBank or in iDSN, respectively. Here, logP means the logarithm of the octanol/water partition coefficient, logS means the logarithm of the solubility.

Supplementary Figure S4 **GO enrichment analyses of drugs in Cluster 28. A**. Dot plot of GO cellular component enrichment results of drugs in Cluster 28. **B**. Dot plot of GO molecular function enrichment results of drugs in Cluster 28. Dot size and color indicate the count of enriched genes in each of the categories and the corresponding significance of enrichment, respectively. Gene ratio represents the ratio of enriched genes to all genes in each of the categories.

Supplementary Figure S5 **Community analysis of Cluster 3**. **A.–D.** Dot plots of KEGG pathway (A), GO biological process (B), GO cellular component (C), and GO molecular function (D) enrichment. Dot size and color indicate the count of enriched genes in each of the categories and the corresponding significance of enrichment, respectively. Gene ratio represents the ratio of enriched genes to all genes in each of the categories. GO IDs are presented in panel B for simplicity. The list of corresponding GO term names associated with these IDs can be found in Table S9. **E**. ChEBI Ontology enrichment results for structure terms. **F**. ChEBI Ontology enrichment result for role terms. Nodes indicate the enriched ChEBI terms. The lower the node transparency is, the more significantly the term is enriched. Arrow proceeds from child to parent term. **G**. Physicochemical feature deviation of drugs in Cluster 3. Red and blue bars represent the indicated drug feature in Cluster 3 that are higher or lower than the average of all drugs in DrugBank database, respectively.

Supplementary Figure S6 **Performance of the iDSN based on superclass data from DrugBank**. **A**. SOR for DSN-S, DSN-C, DSN-T, and iDSN. A bin composed of 3000 drug pairs was slid from the top to the bottom of the drug pairs list with a step size of 100. SOR was calculated for each bin and that for the first 320 bins is plotted here. **B**. Superclass NMIs for DSNs. SOR, superclass overlap rate.

Supplementary Figure S7 **NMIs among DSNs and distributions of DSN-S, DSN-C, and DSN-T**. **A**. The DSN-S, DSN-C, and DSN-T are single-property DSNs. The DSN-S&C, DSN-S&T, and DSN-C&T correspond to fused networks based on two properties. The iDSN is an integrated network based on all three properties. The color of each block corresponds to the NMI of the two networks. **B**. Distributions of DSN-S. **C**. Distributions of DSN-C. **D**. Distributions of DSN-T.

Supplementary Figure S8 **Performance comparison of PIMD and other methods that use 3D structure, expression profiles, and PPI network information**. PIMD outperforms other methods for DSN clustering. Detailed description on other methods can be found in File S1.
