# Supplementary material for: PIMD: An Integrative Approach for Drug Repositioning Using Multiple Characterization Fusion
Source: Genomics Proteomics Bioinformatics. 2020 Oct 17;18(5):565–81. doi: 10.1016/j.gpb.2018.10.012 (PMC8377380; doi:10.1016/j.gpb.2018.10.012)
Supplement: Supplementary File S1 — Drug similarity measurements. [file mmc1.docx]

**File S1 Drug similarity measurements**

**Drug similarity based on 3D structure**

We extracted 48 3D structure descriptors of each drug, which were quantitated based on the Spectrophore. Spectrophore is a kind of fingerprint that can encode the 3D structure of a molecule [[1](#_ENREF_1)]. We computed similarity based on 3D structure between drugs $d$ and $d^{'}$ as the Pearson correlation coefficient of the 3D structure descriptors.

**Drug similarity based on expression profiles**

Drug expression profiles data were retrieved from the Library of Integrated Network-based Cellular Signatures (LINCS) program (<http://lincscloud.org/l1000-data/>). We obtained lists of genes ranked according to their differential expression. All of ranked lists of genes corresponding to the same drug is merged [[2](#_ENREF_2)]. Then we got a single Prototype Ranked List (PRL) of genes for each drug. We selected top-ranked 250 genes and bottom-ranked 250 genes of each PRL as gene signature. Drug $d$ has a enrichment score with respect to the PRL of drug $d^{'}$:

$${ES}_{d,d^{'}}=\frac{{ES}^{up}-{ES}^{down}}{2}$$

${ES}^{up}$ and ${ES}^{down}$ are the enrichment scores of the drug $d$ gene signature (the up-regulated genes and the down-regulated genes, respectively) with respect to the PRL of drug $d^{'}$. The enrichment score is computed using Gene Set Enrichment Analysis (GSEA) [[3](#_ENREF_3)]. Similarly, drug $d^{'}$ has a enrichment score with respect to the PRL of drug $d$. Then similarity based on expression profiles between two drugs $d$ and $d^{'}$ was calculated as follows [[2](#_ENREF_2)] :

$$S_{d,d^{'}}^{expression}=\frac{{ES}_{d,d^{'}}+{ES}_{d^{'},d}}{2}$$

**Drug similarity based on protein–protein interaction network**

We use Dijkstra's algorithm to calculate the shortest paths of drug–target pairs in protein–protein interaction (PPI) network. The PPI network is compiled from the literature [[4](#_ENREF_4)]. It integrates PPI networks from 7 sources and contains 141,296 pairs of relationships between 13,460 proteins. We computed similarity based on PPI network between drug targets $t$ and $t^{'}$ as follows:

$$S_{t,t^{'}}^{PPI}={Ae}^{-bD\left( t,t^{'} \right)}$$

$D(t,t^{'})$ represents the distances between $t$ and $t^{'}$. Here we set $A=0.9 \times e$, $b=1$, recommended by Perlman *et al*. [[5](#_ENREF_5)]. Then, similarity based on PPI network between drugs $d$ and $d^{'}$ was computed as follows:

| $S_{d,d^{'}}^{PPI}=\frac{1}{\left\vert P\left( d \right) \right\vert\times\left\vert P\left( d^{'} \right) \right\vert}\sum_{i=1}^{\left\vert P\left( d \right) \right\vert} \sum_{j=1}^{\left\vert P\left( d^{'} \right) \right\vert} \frac{S_{t,t^{'}}^{PPI}}{\sqrt{S_{t,t}^{PPI}}\sqrt{S_{t^{'},t^{'}}^{PPI}}}$ |
| --- |

$P\left( d \right)$ represents a target set of drug $d$, $P_{i}\left( d \right)$ is the $i$th target of drug $d$, and $\left| P\left( d \right) \right|$ is the size of the target set of drug $d$.

**References**

[1] O’Boyle NM, Banck M, James CA, Morley C, Vandermeersch T, Hutchison GR. Open babel: An open chemical toolbox. J Cheminform 2011;3:33.

[2] Iorio F, Bosotti R, Scacheri E, Belcastro V, Mithbaokar P, Ferriero R, *et al*. Discovery of drug mode of action and drug repositioning from transcriptional responses. Proc Natl Acad Sci U S A 2010;107:14621–6.

[3] Subramanian A, Tamayo P, Mootha VK, Mukherjee S, Ebert BL, Gillette MA, *et al*. Gene set enrichment analysis: a knowledge-based approach for interpreting genome-wide expression profiles. Proc Natl Acad Sci U S A 2005;102:15545–50.

[4] Menche J, Sharma A, Kitsak M, Ghiassian SD, Vidal M, Loscalzo J, *et al*. Uncovering disease-disease relationships through the incomplete interactome. Science 2015;347:1257601.

[5] Perlman L, Gottlieb A, Atias N, Ruppin E, Sharan R. Combining drug and gene similarity measures for drug-target elucidation. J Comput Biol 2011;18:133–45.
