# Supplementary figures and images for: PIMD: An Integrative Approach for Drug Repositioning Using Multiple Characterization Fusion
Source: Genomics Proteomics Bioinformatics. 2020 Oct 17;18(5):565–81. doi: 10.1016/j.gpb.2018.10.012 (PMC8377380; doi:10.1016/j.gpb.2018.10.012)

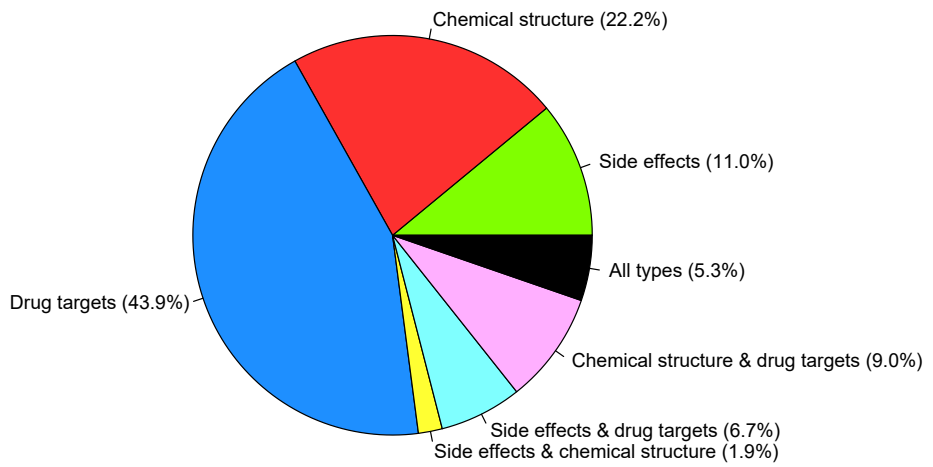

Supplement: Supplementary Figure S2 — Data type contribution analysis for the edges inside the cluster. In the iDSN, within-cluster edges are supported by all types of data analyzed. Among the three single drug properties, the relative contribution of drug target-based data is the greatest. [file mmc3.pdf]

## A Dot plot of GO cellular component enrichment

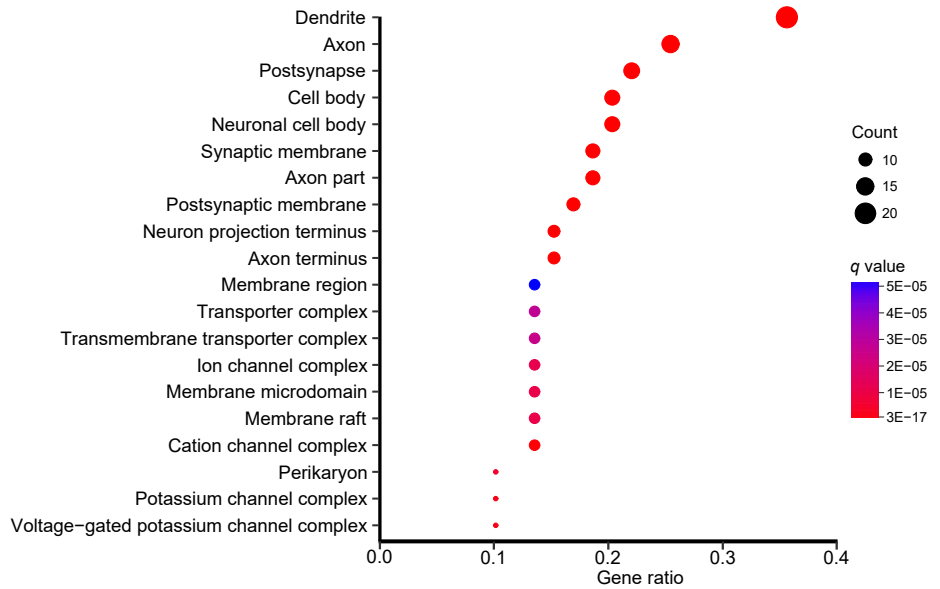

## B Dot plot of GO molecular function enrichment

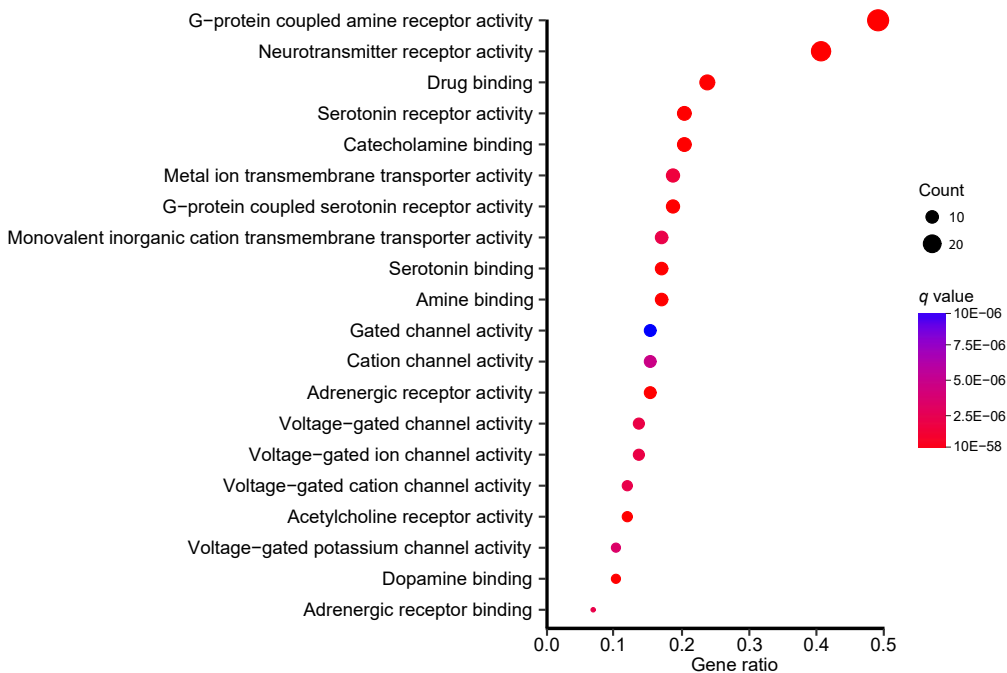

Supplement: Supplementary Figure S4 — GO enrichment analyses of drugs in Cluster 28. A. Dot plot of GO cellular component enrichment results of drugs in Cluster 28. B. Dot plot of GO molecular function enrichment results of drugs in Cluster 28. Dot size and color indicate the count of enriched genes in each of the categories and the corresponding significance of enrichment, respectively. Gene ratio represents the ratio of enriched genes to all genes in each of the categories. [file mmc5.pdf]

**A**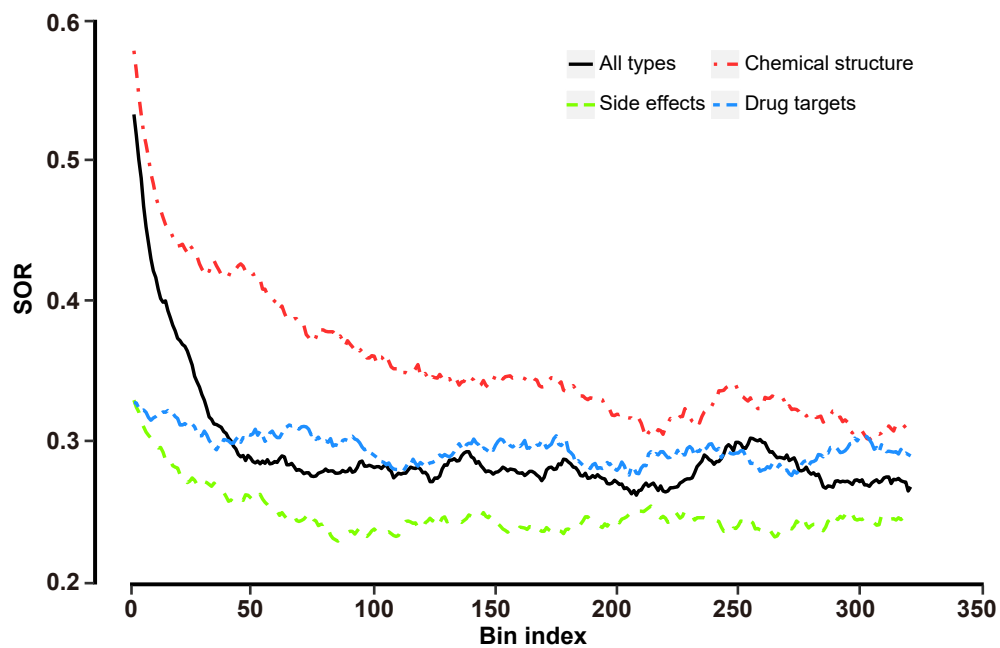**B**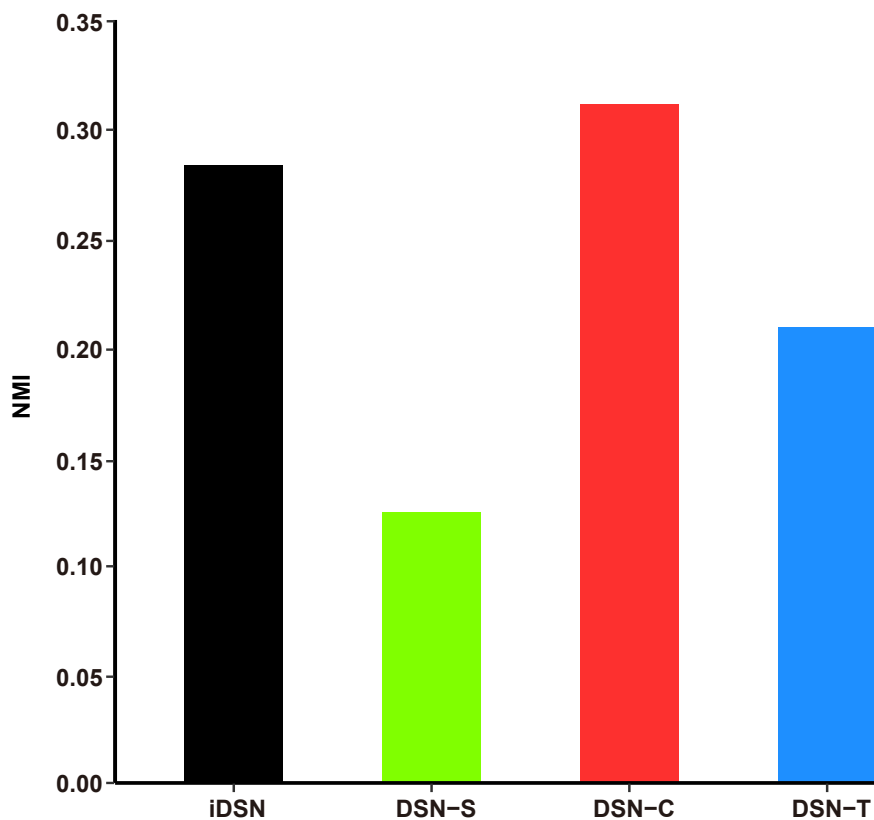

Supplement: Supplementary Figure S6 — Performance of the iDSN based on superclass data from DrugBank. A. SOR for DSN-S, DSN-C, DSN-T, and iDSN. A bin composed of 3000 drug pairs was slid from the top to the bottom of the drug pairs list with a step size of 100. SOR was calculated for each bin and that for the first 320 bins is plotted here. B. Superclass NMIs for DSNs. SOR, superclass overlap rate. [file mmc7.pdf]
