# Supplementary material for: PIMD: An Integrative Approach for Drug Repositioning Using Multiple Characterization Fusion
Source: Genomics Proteomics Bioinformatics. 2020 Oct 17;18(5):565–81. doi: 10.1016/j.gpb.2018.10.012 (PMC8377380; doi:10.1016/j.gpb.2018.10.012)
Supplement: Supplementary Figure S3 — Series of enrichment analyses. For each property (14 physicochemical features),we determined the deviation of its mean value in each cluster from that for all drugs in DrugBank (A) or the mean value for all the 593 drugs in iDSN (B). Red and blue blocks represent features in the cluster that are higher and lower than the average of all drugs in DrugBank or in iDSN, respectively. C. ADMET enrichment analysis. A total of 18 ADMET property terms were extracted from DrugBank. Each property contains two condition terms. We examined whether drugs in the same cluster tend to have the same ADMET property. The higher the ES is, the deeper the color is. D. Enrichment landscape of “target GO cellular component. The deeper the color is, the more significant the enrichment is. Common cellular component themes shared by multiple clusters are boxed with names provided in the plots. E. Enrichment landscape of target GO molecular function. F. Enrichment landscape of target classes. The higher the ES is, the deeper the color is. Target class: LGIC, ligand-gated ion channels; GPCR, G protein-coupled receptors; VGIC, voltage-gated ion channels; NHR, nuclear hormone receptor; Other IC, other ion channels. For each property (62 chemical descriptors), we determined the deviation of its mean value for drugs in each cluster from the mean value for all drugs in DrugBank (G) and the mean value for all the 593 drugs in iDSN (H). Red and blue blocks represent features in the cluster that are higher and lower than average of all drugs in DrugBank or in iDSN, respectively. Here, logP means the logarithm of the octanol/water partition coefficient, logS means the logarithm of the solubility. [file mmc4.pdf]

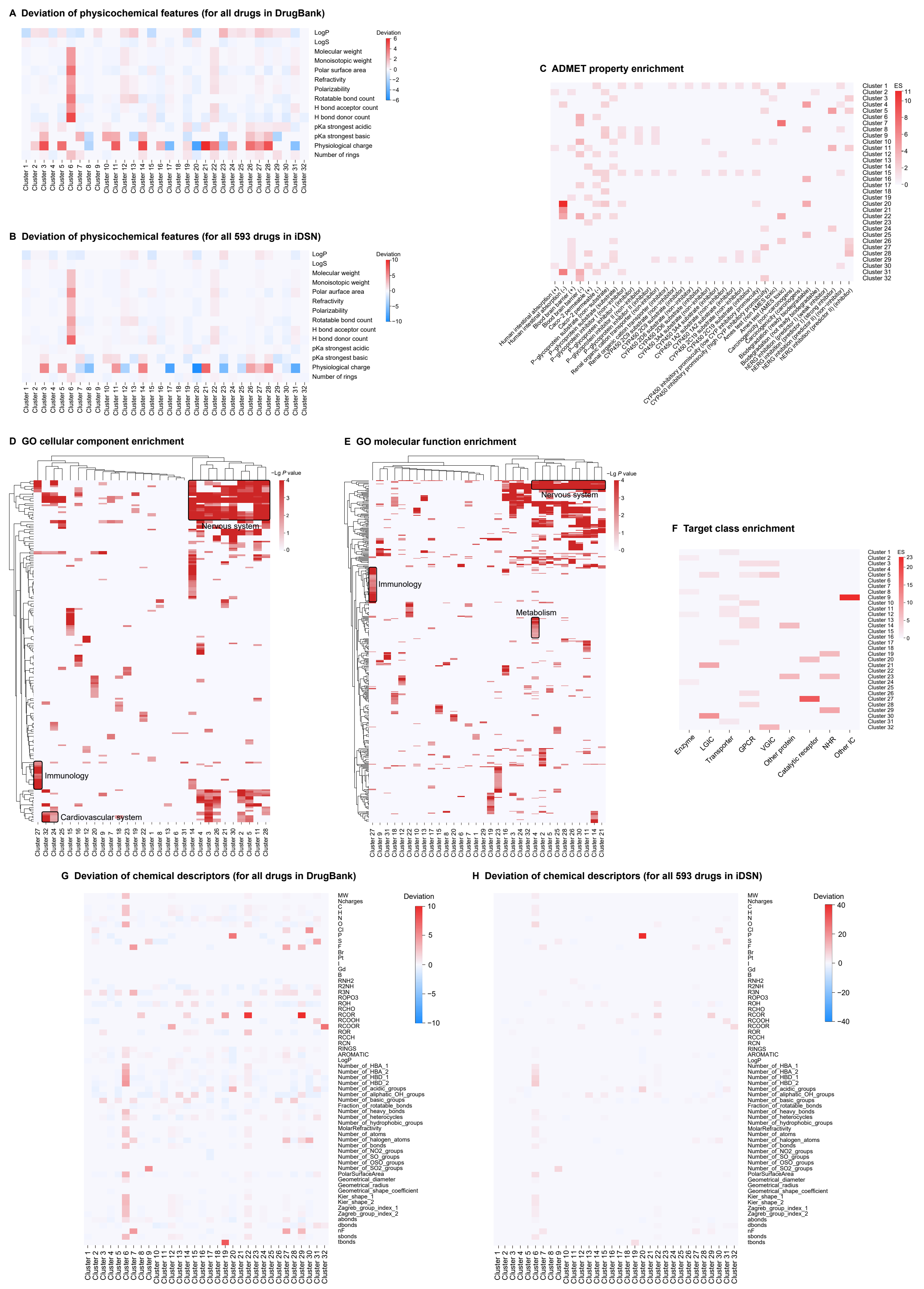
A Deviation of physicochemical features (for all drugs in DrugBank)
LogP
LogS
Molecular weight
Monoisotopic weight
Polar surface area
Refractivity
Polarizability
Rotatable bond count
H bond acceptor count
H bond donor count
pKa strongest acidic
pKa strongest basic
Physiological charge
Number of rings
Deviation
B Deviation of physicochemical features (for all 593 drugs in iDSN)
Polar aurface area
C ADMET property enrichment
ES
Human intestinal absorption (+)
Human intestinal absorption (-)
Blood brain barrier (+)
Blood brain barrier (-)
Caco-2 permeable (+)
Caco-2 permeable (-)
P-glycoprotein substrate (substrate)
P-glycoprotein substrate (non-substrate)
P-glycoprotein inhibitor I (inhibitor)
P-glycoprotein inhibitor I (non-inhibitor)
P-glycoprotein inhibitor II (inhibitor)
P-glycoprotein inhibitor II (non-inhibitor)
Renal organic cation transporter (inhibitor)
Renal organic cation transporter (non-inhibitor)
CYP450 inhibitory promiscuity (low CYP inhibitory promiscuity)
CYP450 inhibitory promiscuity (high CYP inhibitory promiscuity)
Ames test (AMES toxic)
Ames test (non AMES toxic)
Carcinogenicity (carcinogens)
Carcinogenicity (non-carcinogens)
Biodegradation (ready biodegradable)
Biodegradation (not ready biodegradable)
hERG inhibition (predictor I) (weak inhibitor)
hERG inhibition (predictor I) (strong inhibitor)
hERG inhibition (predictor II) (inhibitor)
hERG inhibition (predictor II) (non-inhibitor)
D GO cellular component enrichment
−Lg P value
Nervous system
Immunology
Cardiovascular system
E GO molecular function enrichment
Nervous system
Immunology
Metabolism
F Target class enrichment
Enzyme
LGIC
Transporter
GPCR
VGIC
Other protein
Catalytic receptor
NHR
Other IC
G Deviation of chemical descriptors (for all drugs in DrugBank)
MW
Ncharges
C
H
N
O
Cl
P
S
F
Br
Pt
I
Gd
B
RNH2
R2NH
R3N
ROPO3
ROH
RCHO
RCOR
RCOOH
RCOOR
ROR
RCCH
RCN
RINGS
AROMATIC
LogP
Number_of_HBA_1
Number_of_HBA_2
Number_of_HBD_1
Number_of_HBD_2
Number_of_acidic_groups
Number_of_aliphatic_OH_groups
Number_of_basic_groups
Fraction_of_rotatable_bonds
Number_of_heavy_bonds
Number_of_heterocycles
Number_of_hydrophobic_groups
MolarRefractivity
Number_of_atoms
Number_of_halogen_atoms
Number_of_bonds
Number_of_NO2_groups
Number_of_SO_groups
Number_of_OSO_groups
Number_of_SO2_groups
PolarSurfaceArea
Geometrical_diameter
Geometrical_radius
Geometrical_shape_coefficient
Kier_shape_1
Kier_shape_2
Zagreb_group_index_1
Zagreb_group_index_2
abonds
dbonds
nF
sbonds
tbonds
H Deviation of chemical descriptors (for all 593 drugs in iDSN)
