# Supplementary material for: PIMD: An Integrative Approach for Drug Repositioning Using Multiple Characterization Fusion
Source: Genomics Proteomics Bioinformatics. 2020 Oct 17;18(5):565–81. doi: 10.1016/j.gpb.2018.10.012 (PMC8377380; doi:10.1016/j.gpb.2018.10.012)
Supplement: Supplementary Figure S5 — Community analysis of Cluster 3. A.–D. Dot plots of KEGG pathway (A), GO biological process (B), GO cellular component (C), and GO molecular function (D) enrichment. Dot size and color indicate the count of enriched genes in each of the categories and the corresponding significance of enrichment, respectively. Gene ratio represents the ratio of enriched genes to all genes in each of the categories. GO IDs are presented in panel B for simplicity. The list of corresponding GO term names associated with these IDs can be found in Table S9. E. ChEBI Ontology enrichment results for structure terms. F. ChEBI Ontology enrichment result for role terms. Nodes indicate the enriched ChEBI terms. The lower the node transparency is, the more significantly the term is enriched. Arrow proceeds from child to parent term. G. Physicochemical feature deviation of drugs in Cluster 3. Red and blue bars represent the indicated drug feature in Cluster 3 that are higher or lower than the average of all drugs in DrugBank database, respectively. [file mmc6.pdf]

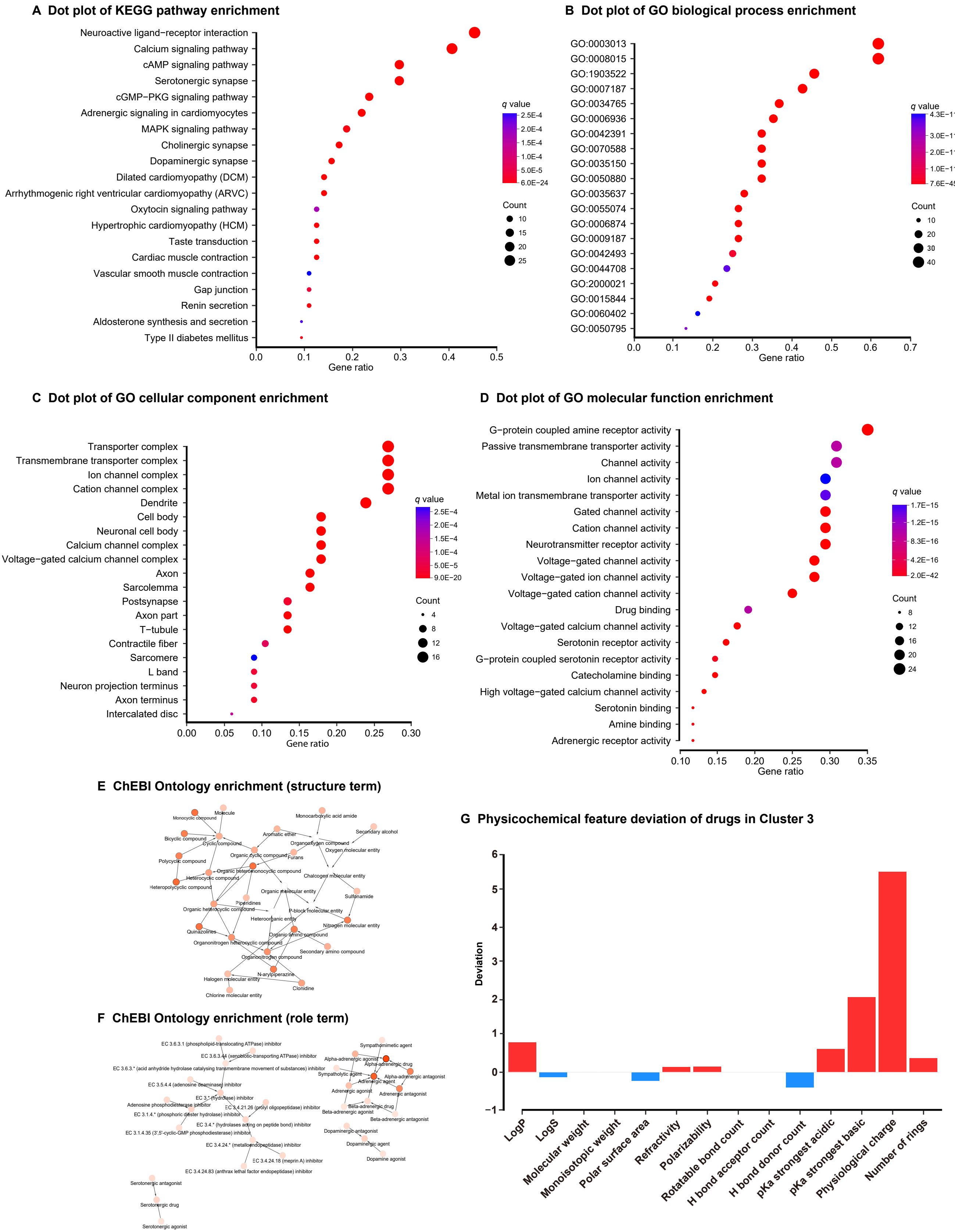
A Dot plot of KEGG pathway enrichment
Neuroactive ligand−receptor interaction
Calcium signaling pathway
cAMP signaling pathway
Serotonergic synapse
cGMP−PKG signaling pathway
Adrenergic signaling in cardiomyocytes
MAPK signaling pathway
Cholinergic synapse
Dopaminergic synapse
Dilated cardiomyopathy (DCM)
Arrhythmogenic right ventricular cardiomyopathy (ARVC)
Oxytocin signaling pathway
Hypertrophic cardiomyopathy (HCM)
Taste transduction
Cardiac muscle contraction
Vascular smooth muscle contraction
Gap junction
Renin secretion
Aldosterone synthesis and secretion
Type II diabetes mellitus
0.0 0.1 0.2 0.3 0.4 0.5
Gene ratio
q value
2.5E-4
2.0E-4
1.5E-4
1.0E-4
5.0E-5
6.0E−24
Count
10
15
20
25
B Dot plot of GO biological process enrichment
GO:0003013
GO:0008015
GO:1903522
GO:0007187
GO:0034765
GO:0006936
GO:0042391
GO:0070588
GO:0035150
GO:0050880
GO:0035637
GO:0055074
GO:0006874
GO:0009187
GO:0042493
GO:0044708
GO:2000021
GO:0015844
GO:0060402
GO:0050795
0.0 0.1 0.2 0.3 0.4 0.5 0.6 0.7
Gene ratio
q value
4.3E−11
3.0E−11
2.0E−11
1.0E−11
7.6E−45
Count
10
20
30
40
C Dot plot of GO cellular component enrichment
Transporter complex
Transmembrane transporter complex
Ion channel complex
Cation channel complex
Dendrite
Cell body
Neuronal cell body
Calcium channel complex
Voltage−gated calcium channel complex
Axon
Sarcolemma
Postsynapse
Axon part
T−tubule
Contractile fiber
Sarcomere
L band
Neuron projection terminus
Axon terminus
Intercalated disc
0.00 0.05 0.10 0.15 0.20 0.25 0.30
Gene ratio
q value
2.5E−4
2.0E−4
1.5E−4
1.0E−4
5.0E−5
9.0E−20
Count
4
8
12
16
D Dot plot of GO molecular function enrichment
G−protein coupled amine receptor activity
Passive transmembrane transporter activity
Channel activity
Ion channel activity
Metal ion transmembrane transporter activity
Gated channel activity
Cation channel activity
Neurotransmitter receptor activity
Voltage−gated channel activity
Voltage−gated ion channel activity
Voltage−gated cation channel activity
Drug binding
Voltage−gated calcium channel activity
Serotonin receptor activity
G−protein coupled serotonin receptor activity
Catecholamine binding
High voltage−gated calcium channel activity
Serotonin binding
Amine binding
Adrenergic receptor activity
0.10 0.15 0.20 0.25 0.30 0.35
Gene ratio
q value
1.7E−15
1.2E−15
8.3E−16
4.2E−16
2.0E−42
Count
8
12
16
20
24
E ChEBI Ontology enrichment (structure term)
Molecule
Monocyclic compound
Monocarboxylic acid amide
Secondary alcohol
Aromatic ether
Bicyclic compound
Cyclic compound
Organooxygen compound
Oxygen molecular entity
Organic cyclic compound
Furans
Polycyclic compound
Organic heteromonocyclic compound
Heterocyclic compound
Chalcogen molecular entity
Heteropolycyclic compound
Organic molecular entity
Sulfonamide
Piperidines
Organic heterocyclic compound
P-block molecular entity
Heteroorganic entity
Nitrogen molecular entity
Quinazolines
Organic amino compound
Organonitrogen heterocyclic compound
Secondary amino compound
Organonitrogen compound
N-arylpiperazine
Halogen molecular entity
Clonidine
Chlorine molecular entity
F ChEBI Ontology enrichment (role term)
EC 3.6.3.1 (phospholipid-translocating ATPase) inhibitor
EC 3.6.3.44 (xenobiotic-transporting ATPase) inhibitor
EC 3.6.3.* (acid anhydride hydrolase catalysing transmembrane movement of substances) inhibitor
EC 3.5.4.4 (adenosine deaminase) inhibitor
EC 3.* (hydrolase) inhibitor
Adenosine phosphodiesterase inhibitor
EC 3.4.21.26 (prolyl oligopeptidase) inhibitor
EC 3.1.4.* (phosphoric diester hydrolase) inhibitor
EC 3.4.* (hydrolases acting on peptide bond) inhibitor
EC 3.1.4.35 (3',5'-cyclic-GMP phosphodiesterase) inhibitor
EC 3.4.24.* (metalloendopeptidase) inhibitor
EC 3.4.24.18 (meprin A) inhibitor
EC 3.4.24.83 (anthrax lethal factor endopeptidase) inhibitor
Sympathomimetic agent
Alpha-adrenergic agonist
Alpha-adrenergic drug
Sympatholytic agent
Alpha-adrenergic antagonist
Adrenergic agent
Adrenergic agonist
Adrenergic antagonist
Beta-adrenergic drug
Beta-adrenergic agonist
Beta-adrenergic antagonist
Dopaminergic antagonist
Dopaminergic agent
Dopamine agonist
Serotonergic antagonist
Serotonergic drug
Serotonergic agonist
G Physicochemical feature deviation of drugs in Cluster 3
Deviation
6
5
4
3
2
1
0
–1
LogP
LogS
Molecular weight
Monoisotopic weight
Polar surface area
Refractivity
Polarizability
Rotatable bond count
H bond acceptor count
H bond donor count
pKa strongest acidic
pKa strongest basic
Physiological charge
Number of rings
