# Supplementary material for: PIMD: An Integrative Approach for Drug Repositioning Using Multiple Characterization Fusion
Source: Genomics Proteomics Bioinformatics. 2020 Oct 17;18(5):565–81. doi: 10.1016/j.gpb.2018.10.012 (PMC8377380; doi:10.1016/j.gpb.2018.10.012)
Supplement: Supplementary Table S1 [file mmc10.docx]

**Table S1 Contribution of each data type or combination of different data types for each cluster**

| Cluster | Drug side effects | Drug chemical structure | Drug targets | Drug side effects & chemical structure | Drug side effects & targets | Drug chemical structure & targets | All data types |
| --- | --- | --- | --- | --- | --- | --- | --- |
| 1 | 0.19883 | 0.233918 | 0.280702 | 0.040936 | 0.146199 | 0.035088 | 0.064327 |
| 2 | 0.072055 | 0.089599 | 0.615915 | 0.006892 | 0.078947 | 0.093985 | 0.042607 |
| 3 | 0.219373 | 0.205128 | 0.31339 | 0.045584 | 0.079772 | 0.074074 | 0.062678 |
| 4 | 0.04955 | 0.055556 | 0.662162 | 0.001502 | 0.093093 | 0.097598 | 0.040541 |
| 5 | 0.152381 | 0.238095 | 0.247619 | 0.047619 | 0.071429 | 0.128571 | 0.114286 |
| 6 | 0 | 0.944444 | 0 | 0.055556 | 0 | 0 | 0 |
| 7 | 0.027778 | 0.75 | 0.083333 | 0.027778 | 0 | 0.111111 | 0 |
| 8 | 0.019048 | 0.12381 | 0.72381 | 0 | 0.047619 | 0.066667 | 0.019048 |
| 9 | 0.128205 | 0.474359 | 0.102564 | 0.038462 | 0.076923 | 0.051282 | 0.128205 |
| 10 | 0.051471 | 0.301471 | 0.485294 | 0.022059 | 0.014706 | 0.073529 | 0.051471 |
| 11 | 0.241107 | 0.181818 | 0.312253 | 0.027668 | 0.011858 | 0.15415 | 0.071146 |
| 12 | 0.242165 | 0.301994 | 0.159544 | 0.031339 | 0.082621 | 0.094017 | 0.088319 |
| 13 | 0 | 0.928571 | 0 | 0 | 0 | 0.071429 | 0 |
| 14 | 0.087912 | 0.582418 | 0.263736 | 0.010989 | 0.021978 | 0.010989 | 0.021978 |
| 15 | 0.228571 | 0.609524 | 0.038095 | 0.038095 | 0 | 0.047619 | 0.038095 |
| 16 | 0.138889 | 0.416667 | 0.222222 | 0.027778 | 0.055556 | 0.111111 | 0.027778 |
| 17 | 0.071146 | 0.43083 | 0.221344 | 0.039526 | 0.063241 | 0.083004 | 0.090909 |
| 18 | 0.071429 | 0.285714 | 0.464286 | 0 | 0.035714 | 0.071429 | 0.071429 |
| 19 | 0.264706 | 0.507353 | 0.066176 | 0.044118 | 0.058824 | 0.022059 | 0.036765 |
| 20 | 0.142857 | 0.571429 | 0.107143 | 0 | 0 | 0.178571 | 0 |
| 21 | 0.357143 | 0.107143 | 0.428571 | 0 | 0.035714 | 0.071429 | 0 |
| 22 | 0.111111 | 0.466667 | 0.2 | 0.044444 | 0.066667 | 0.044444 | 0.066667 |
| 23 | 0.205128 | 0.166667 | 0.358974 | 0 | 0.051282 | 0.166667 | 0.051282 |
| 24 | 0.057576 | 0.068687 | 0.617172 | 0.005051 | 0.09596 | 0.10404 | 0.051515 |
| 25 | 0.07619 | 0.07619 | 0.6 | 0.009524 | 0.085714 | 0.095238 | 0.057143 |
| 26 | 0.252632 | 0.231579 | 0.252632 | 0.021053 | 0.015789 | 0.173684 | 0.052632 |
| 27 | 0.134503 | 0.380117 | 0.210526 | 0.087719 | 0.011696 | 0.070175 | 0.105263 |
| 28 | 0.060606 | 0.316017 | 0.415584 | 0.017316 | 0.060606 | 0.095238 | 0.034632 |
| 29 | 0.033333 | 0.766667 | 0.125 | 0.025 | 0 | 0.025 | 0.025 |
| 30 | 0.035573 | 0.320158 | 0.533597 | 0.007905 | 0.023715 | 0.063241 | 0.01581 |
| 31 | 0.190476 | 0.52381 | 0.057143 | 0.066667 | 0 | 0.07619 | 0.085714 |
| 32 | 0.145455 | 0.654545 | 0.109091 | 0 | 0.072727 | 0 | 0.018182 |

*Note:* Values in the table indicate the contributions of various data types or their combinations, which were calculated as described in data type contribution in Methods section.
