# Supplementary material for: PIMD: An Integrative Approach for Drug Repositioning Using Multiple Characterization Fusion
Source: Genomics Proteomics Bioinformatics. 2020 Oct 17;18(5):565–81. doi: 10.1016/j.gpb.2018.10.012 (PMC8377380; doi:10.1016/j.gpb.2018.10.012)
Supplement: Supplementary Table S9 [file mmc18.docx]

**Table S9 ID and name of GO biological process terms enriched in Cluster 28 or Cluster 3**

| GO ID | GO term name |
| --- | --- |
| GO:0007187 | G-protein coupled receptor signaling pathway, coupled to cyclic nucleotide second messenger |
| GO:0008015 | Blood circulation |
| GO:0044057 | Regulation of system process |
| GO:0035150 | Regulation of tube size |
| GO:0050880 | Regulation of blood vessel size |
| GO:0043410 | Positive regulation of MAPK cascade |
| GO:0072507 | Divalent inorganic cation homeostasis |
| GO:0044708 | Behavior |
| GO:0042493 | Response to drug |
| GO:0034765 | Regulation of ion transmembrane transport |
| GO:0006874 | Cellular calcium ion homeostasis |
| GO:0006939 | Smooth muscle contraction |
| GO:0015844 | Monoamine transport |
| GO:0050890 | Cognition |
| GO:0009187 | Cyclic nucleotide metabolic process |
| GO:0042220 | Response to cocaine |
| GO:0031279 | Regulation of cyclase activity |
| GO:1905145 | Cellular response to acetylcholine |
| GO:1905144 | Response to acetylcholine |
| GO:0095500 | Acetylcholine receptor signaling pathway |
| GO:0003013 | Circulatory system process |
| GO:1903522 | Regulation of blood circulation |
| GO:0006936 | Muscle contraction |
| GO:0042391 | Regulation of membrane potential |
| GO:0070588 | Calcium ion transmembrane transport |
| GO:0035150 | Regulation of tube size |
| GO:0035637 | Multicellular organismal signaling |
| GO:0055074 | Calcium ion homeostasis |
| GO:2000021 | Ion homeostasis |
| GO:0060402 | Calcium ion transport into cytosol |
| GO:0050795 | Regulation of behavior |

*Note:* The enriched GO IDs for Cluster 28 and Cluster 3 were presented in Figure 6B and Figure S5B, respectively.
